# Research on deformation characteristics and mechanisms of an open pit coal mine landslide event in extremely cold region

**DOI:** 10.1038/s41598-025-27509-5

**Published:** 2025-12-08

**Authors:** Han Du, Yang Wan, Dong Wang, Bo Cao, Guangwei Liu

**Affiliations:** 1 China Coal Research Institute, Beijing, 100013 China; 2 School of Construction Engineering, Shenzhen Polytechnic University, Shenzhen, 518055 China; 3 Department of Hydraulic Engineering, Tsinghua University, Beijing, 100084 China; 4 College of Mining, Liaoning Technical University, Fuxin, 123000 China

**Keywords:** Landslides, Open-pit mine, Extremely cold climate, Failure mechanism, Digital image correlation (DIC), Production scheduling, Natural hazards, Civil engineering

## Abstract

For the sake of heavy rainfall, a landslide occurred at the Baorixile open-pit coal mine, at 13:40 (Beijing time, UTC + 8) on April 30, 2020, in China. The landslide event was about 130 × 10^5^ m^3^ and produced considerable damage in addition to economic loss. Determining the trigger factors and ascertaining unstable slopes and initial displacements – which can be attained by utilizing remote sensing data are pivotal for disaster prevention and risk reduction in open-pit mining areas. An exhaustive integrated methodology was applied. This methodology combined post-hazard investigation, UAV aerial digital photogrammetry interpretation, and geological mapping. It was used to analyze the deformation characteristics and clarify the process and causes.deformation characteristics. The research adopts this approach through back analysis of the Baorixile open-pit coal mine landslide event. Initially, the landslide area was split into four subzones: the rear tension crack zone, the detachment zone at the top, the middle sliding subsidence zone, and the anterior sliding colluvium zone. The analysis suggests that engineering activities, freeze-thawing, and persistent rainfall were the dominant external factors triggering the landslide. Weak intercalated layers in the local topography were the principal inherent factors for the event. The digital image correlation technique reveals each stage in the four-stage hazard chain identified by AL-DIC during the whole failure processing. Finally, the objective of this research is to supply an acquaintance with toppling stage connected to the interplay between the climate and open-pit mining related instabilities.

## Introduction

Rockfalls and landslides, caused by ground subsidence or slope instability processes, are very frequent phenomena during mining and for many years after the cessation of mining^1–5^. Apart from dislocating quantities of mined minerals and increasing production costs, rock mass movements and surface deformation potentially lead to slope failures entailing precarious conditions for personnel, apparatus, and infrastructures in open-pit mining environment^6–9^. The geotechnical slope design process for open-pit mines at this production size is predominantly influenced by risk minimization imperatives. As a result, the resulting slope configurations are often overly conservative due to the necessity of balancing operational risks with economic advantages^10–12^. Exhaustive slope monitoring technologies (e.g. ground-based, airborne-based, and remotely sensed surveying) and modeling programs are regarded as the undeniable fundamental precautionary measures with the aim of assuring open pit mine safety^13–15^.

Slope monitoring techniques can detect small precursor movements of slopes for an extended period ranging from weeks to months prior to instability in open pit mining areas. Monitoring techniques used to warn of impending failures in open-pit mines can be broadly subdivided into two categories^16^: (i) topographic surveying (total stations/prisms, tiltmeters, theodolites, GPS et al.)^17–20^ (ii) subsurface measurement techniques (acoustic emission, inclinometers, `extensometers, time-domain reflectometry et al.)^21–23^. However, during exploitation and reclamation, staffing and budgetary limitations as well as decreased personnel exposure tend to make the afore-mentioned two survey techniques less rewarding. The main drawbacks can be generalized as three following points: (a) firstly, they all require entry to the monitoring surface for establishment and maintenance of apparatus. (b) posteriorly, these techniques generally collect a few separate points on a monitoring surface, hence, fail to provide a spatially distributed description for evaluating the kinematic behavior of the entire mine wall^24–26^. (c) lately, the application of remote sensing has stood out as a cutting-edge technology for real-time slope failure monitoring but its use in the mining sector remains scarce^27–31^. Within this context, recent upgrading in improvement of precise displacement measurement techniques have yielded sturdy and sophisticated devices for slope monitoring and open pit mine administration. For example, displacement monitoring has become a cutting-edge technology for slope monitoring owing to its capability to detect movements with high accuracy, spatial coverage, and frequency of acquisition. Serious slope instabilities are usually accompanied by the gradual development of tension cracks behind the slope crest and measurable displacements. Currently, ocular inspection is the most efficacious action for detecting new cracks and fissures followed by monitoring of their propagation and evolution with the passage of time. Manual qualitative flagging has drawbacks: it is labor-intensive and exposes personnel to unnecessary hazards and risks. More recently, portable wireline extensometer has become the most common method for monitoring movement across tension cracks. The distinct preponderances of the portable wireline extensometer in use are they have a digital readout and can be stored in an electronic data logger and then downloaded to a personal computer.

The failure of slopes in open pit mine are aspects of various foundations such as slope, height, type of lithology, geological factors influencing the stability of rock slopes. Nonetheless, slope failure susceptibility investigation is usually difficult and computationally intensive as well as extensive. Alternatively, for efficient mitigation and management of disasters, certain remote sensing technologies are very effective tools helping in monitoring, assessment, identifying gaps appropriate strategies. Generally, Digital Image Correlation (DIC)^32–35^ photogrammetry technique^36^ has the potential to overcome the above difficulties and perform forceful substitutes for the remote monitoring of open-pit slopes. DIC is an image processing technique that permits the tracking of subsets of unique pixel intensity texture through a sequence of digital images taken from a fixed camera location to a sub-pixel accuracy approaching 0.001 pixels.

Recent advancements in Digital Image Correlation (DIC) techniques, in conjunction with multi-view stereopsis (MVS) algorithms^37,38^, unmanned aerial vehicles (UAVs)^39^, and high-resolution cameras^40^, have facilitated automated 3D surface reconstruction without the necessity for manual ground control points or camera pose estimation^41^. In contrast to conventional photogrammetry (Wolf & Dewitt, 2000), Digital Image Correlation (DIC) autonomously aligns features across many photos and reconstructs scene geometry via nonlinear least-squares minimization (NLS)^42^. This technique has been extensively utilized in geohazard investigations associated with mining, including slope stability research^43^, surface subsidence monitoring^44^, and landslide detection^12^.

**Fig. 1:**
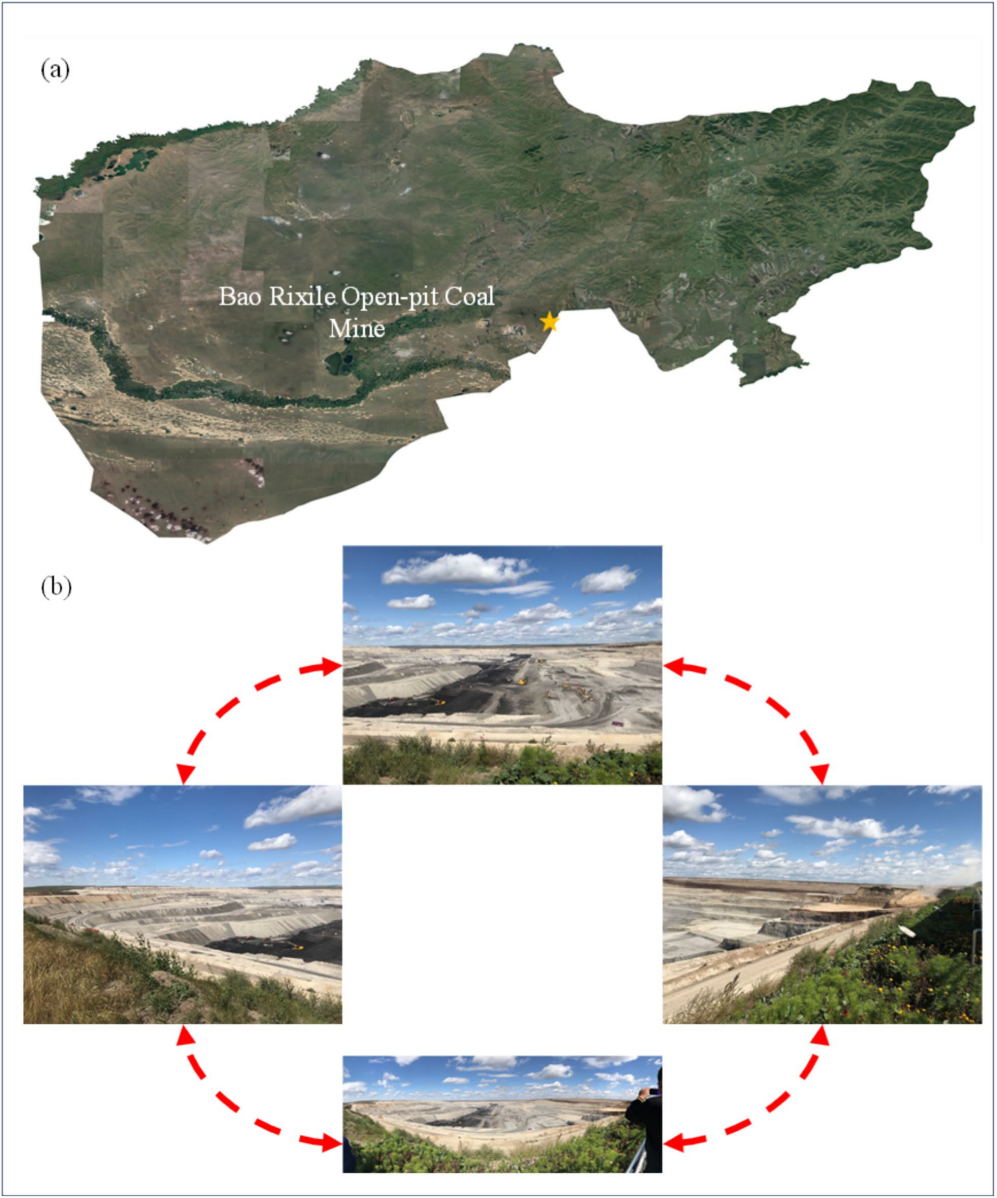
(**a**) Location of the study area. (**b**) Typical photos of Bao Rixile Open-pit Coal Mine failures with unmanned aerial vehicle (UAV) in multiaspects. (Noted, Fig. 1a is made from the cross-platform map browsing software developed by Interactive Map Software Co., Ltd.).

In this study, we analyze the formation mechanism and deformation characteristics of the large-scale landslide that occurred in the Baorixile open pit mine (Fig. 1) after heavy rainfall in April 2020. Particularly, during this event, the work bench of elevation 690 m of landslide trailing edge (pit bottom) between N5474000 to N5472000 (in the horizontal direction) and E40483000 to E40483500 (in vertical direction) survey lines, which elevation line is uplifted comprehensively and the crack increases to form the reverse slope embankment. The large-scale landslide poses a severe threat to the mining plan because the mass movement buried the primary mineable coal seam. We put forth a multidisciplinary methodology that combines DIC photogrammetry, detailed land investigations, and video monitoring through the back analysis of the landslide event to study its kinematics with correlated data.

The research is organized as follows. The case study section describes the slope failure, the study area, the mining operation, and the lithology involved. Then, in the methodology section, we provide a description of the methods and datasets used. Artificial inspection and crack-monitoring data (from December 2019 until the slope failure event) from the monitoring points in the right area allow us to characterize the deformation type and how it changed over time in an open pit mine in a very cold area. In the section on image correlation techniques, this study also tries to use the modified AL-DIC in addition to the standard DIC to look at landslides. This can help fix problems with the standard DIC that are caused by noise from instruments and the atmosphere, as well as problems with spatial and temporal correlations and co-registration errors. It is necessary to understand the failure mechanisms and evolution process of a failure event in open pit mine. This research aims to provide field evidence and methods for choosing the key precautions region and adjusting the engineering econtrol scheme of open pit mining of such catastrophic hazards through scrutinizing regional conditions, temporal and spatial characteristics of mining engineering activities, morphostratigraphy structure, predisposing mechanisms and triggered factors, and the evolution process. This study meticulously analysed the deformation characteristics and mechanisms of the Balzhikhe landslide event by the comprehensive use of remote sensing data, UAV aerial digital photogrammetry, and geological surveys. To thoroughly comprehend the mechanism of landslide occurrence, the study employed back analysis in conjunction with digital image correlation (DIC) technology to monitor and analyse the landslide process across several stages. Subsequently, we will elucidate the specific methodologies and data sets employed in this work.

### Study area

**Fig. 2:**
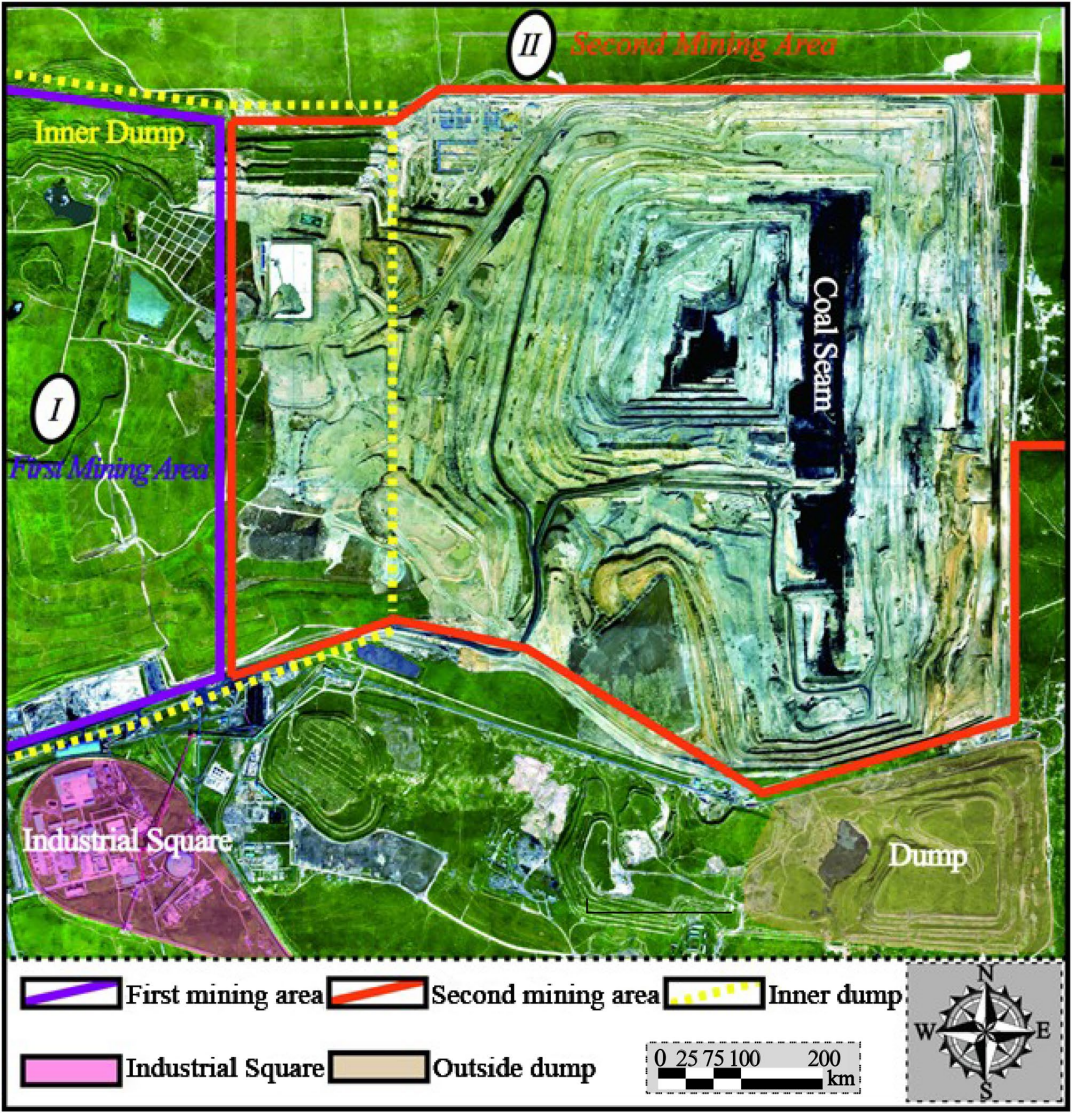
Orthoimages of pre-failure topography and scenario distribution in Baorixile open-pit coal mine (The green part of the image shows that the landscape was restored to grassland after mining) (Noted, Fig. 2 is made is provided by Baorixile Open-pit Coal Mine).

The Baorixile open-pit coal mine (Figs. 1 and 2), located at Prairie Chenbarhu Banner (49°23′41″N, 119°45′33″E) in the north fringe of Hulunbuir city and on the 20.23 km north direction of Hailar district, is one of the largest mines in Inner Mongolia Autonomous Region, China. The mine has an overall mining area of approximately 50.72 km^2^, making it one of the biggest state-owned enterprises in northeastern Inner Mongolia Autonomous, reserving more than 1372.52 million tonnes of coal ore^45^;. Baorixile open pit mine began construction in September 1998 and has now been operating for 20 years (first went into operation in April, 2001). For more than 20 years practically constant operation, this mine consists of five mining areas (mine *I*, *II*, *III*, *IV* and *V*, respectively); *III* mining area with the depth of 130.34 m was turned into a giant pit with the length and width of 3,150 m and 2,450 m. *II* mining area has been regarded as the most active extraction site (Fig. 2). The first mining area, the third mining area and the fourth mining area, as well as the inside and outside dumping sites of towering, have achieved remarkable results under the policy of greening and supporting: covering grassland rotting soil, land reclamation, planting grass and planting trees (as shown in Fig. 2).

### Case study: Baorixile open-pit coal mine slope failure

**Fig. 3:**
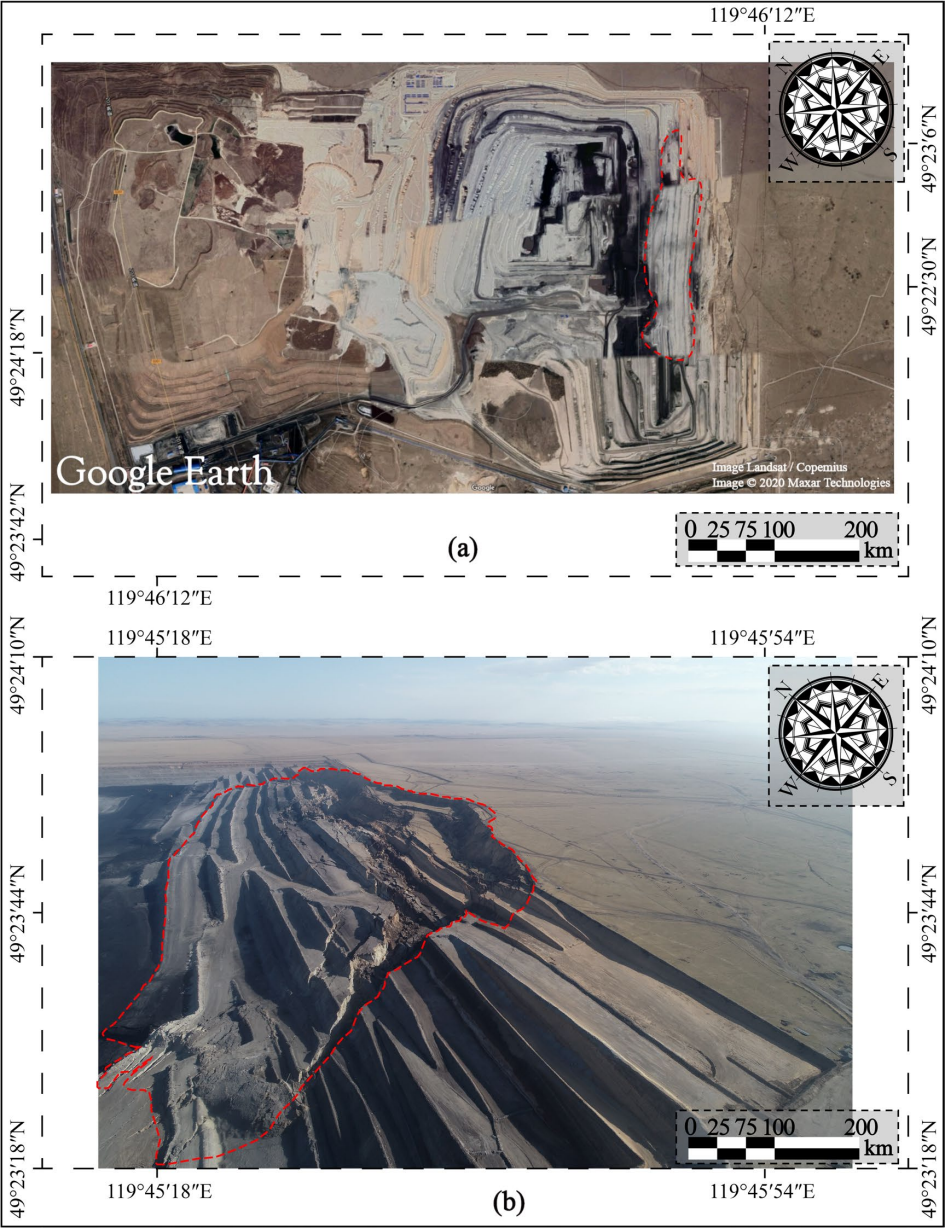
Comprarsion of a pre**-** sliding satellite image from Google Earth (**a**) and post-sliding image taken by UAV (**b**) of the Baorixile open-pit coal mine landslide (Noted, Fig. 3a is made from the Google Earth).

**Fig. 4:**
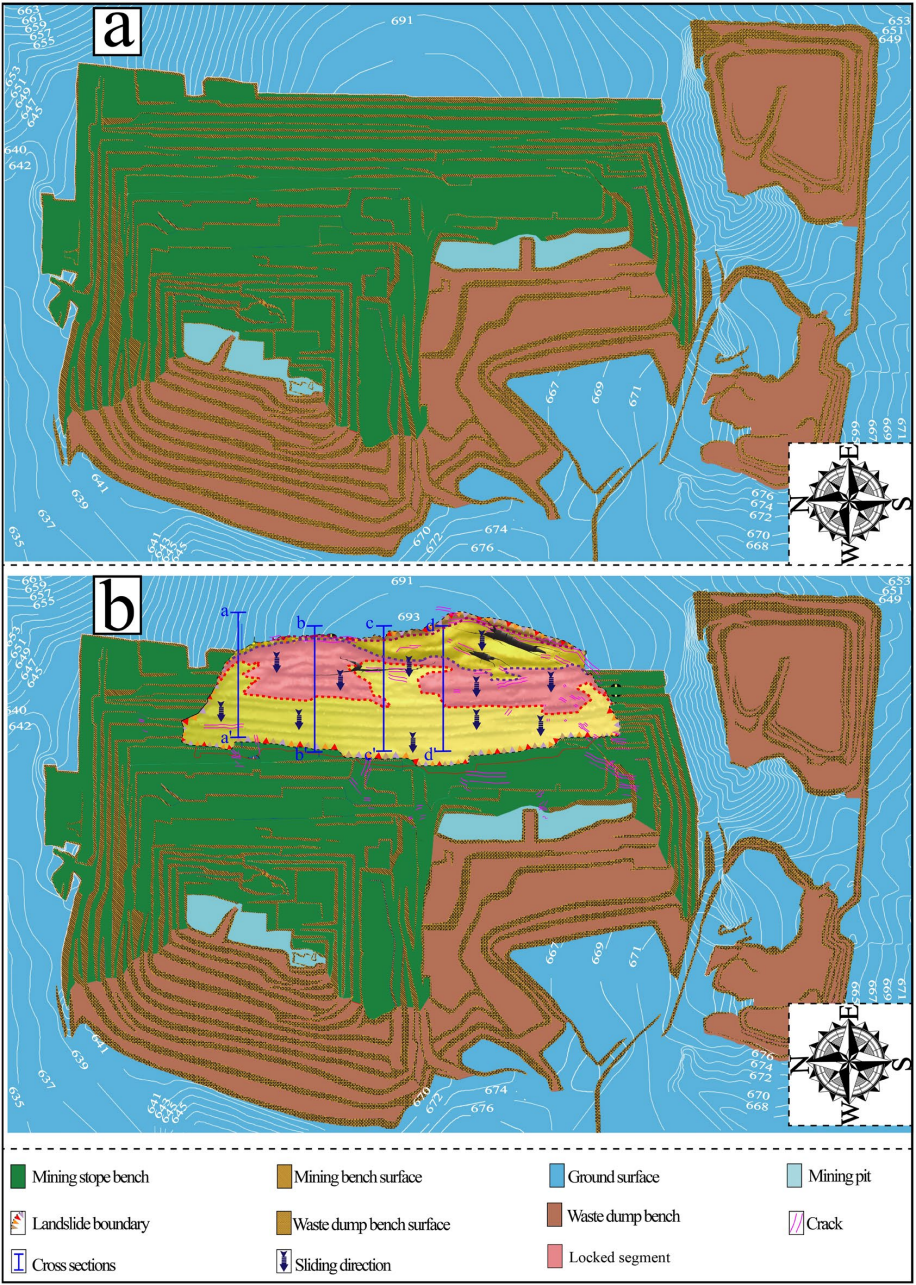
Simplified engineering geological map of the Baorixile landslide. (**a**) Pre-failure. (**b**) Post- failure. (Noted, Generated by the SMcad software V7.0 independently developed by Liaoning Technical University).

**Fig. 5:**
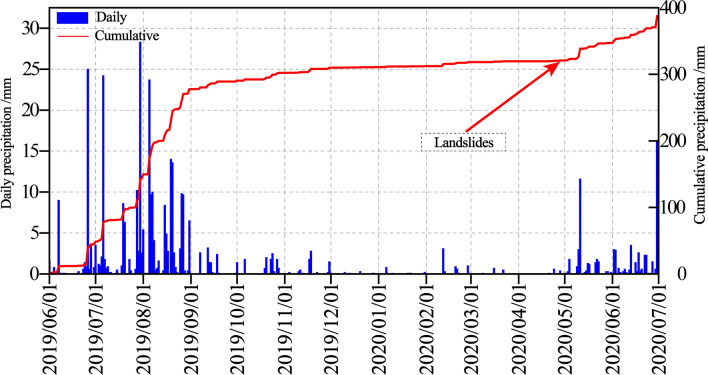
Typical section profile of landslide area: (**a**) Longitudinal profile a-a′; (**b**) Longitudinal profile b-b′; (**c**) Longitudinal profile c–c′; (**d**) Longitudinal profile d-d′.

At ca. 13:40 (Beijing Time, UTC + 8) on April 30, 2020, an unforeseen landslide (N49°23′41″, E119°45′33″) occurred on the east slope of the *II* mining area in Baorixile open-pit coal mine. Figure 3 shows the post-sliding field photo and pre-sliding three-dimensional (3D) view of the Baorixile landslide. The pre-sliding images (Fig. 3a) are from Google Earth, taken on 11 November 2019 with a resolution of 200 m. Figure 3b shows the post sliding aerial photograph of the landslide, taken on May 1, 2020, with a resolution of 200 m, which was obtained within 24 h after the event. According to the basic and original data of the Baorixile open-pit mine and the on-site realistic analysis after the landslide event, we briefly drew the plane diagram of the study area (Fig. 4a and b), so as to better analyze the relevant conditions of the landslide (such as the spatial geometry type and landslide mechanism) in the subsequent research progress. The outline of the landslide has a dustpan-like shape in frontal panoramic view, with a wide anterior profile and broad trailing and front areas (Fig. 4b). By means of geological inspecting and surveying, the length of the slide from crest to toe was practically in an appraisal of 98 m width while the maximum longitudinal length of the slide from north to south was 1800 m thereabouts. It has an estimated volume of 130 × 10^5^ m^3^, covering an area of 464 × 10^3^ m^2^, with a vertical surface subsidence of 28 m. Pursuant to geological investigation and symbolic cross sections (Fig. 5a–d), the probable depth of slip surface is more than 40 m. The elevation of the trailing edge is approximately 695 m, the front edge reaches the bottom of mining pit, the average terrain gradient of the landslide is 16°, the relative elevation difference is up to 150 m. Fortunately, the failure occurred during a shift change, and no mortality or casualties were reported. The landslide caused damage to several pieces of mining apparatus and the roads of transporting waste materials whose construction had been accomplished the preceding year were buried by the moving mass.

**Fig. 6:**
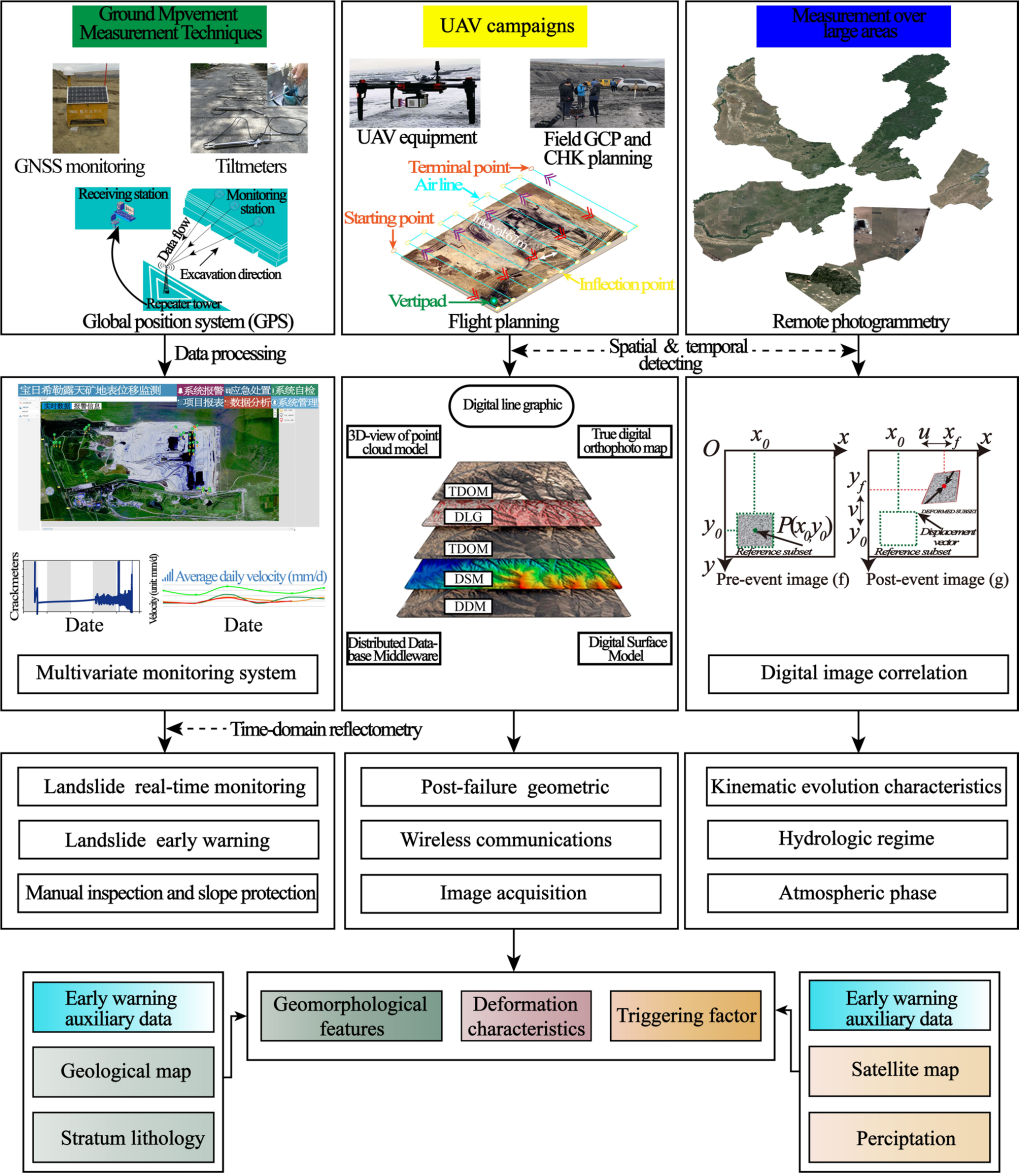
Aerial photo of the landslide after the disaster (Noted, (**a**–**c**) is captured by the authors, Wan Yang and Du Han, helped by provided by Baorixile Open-pit Coal Mine).

Promptly after the failure event, the enterprise and the local government quickly rush to rapid emergency deployment response, and the related datum were acquired, namely, unmanned aerial vehicle (UAV) survey, detailed topography investigations, and deformation observation, which provides valued information and on-site for contingency response plan (Figs. 3b and 6).

### Geomorphology

The Baorixile coalfield is situated in the rural hinterland sector of the Hulun Buir Grassland, whose topography is a slightly undulating high plain in macro display which is attributed to typical half-graben landscape of Chen Qi. From the overall structural characteristics of coalfield, it is located in the middle north of the Hailar subsidence zone, the third subsidence belt of Neocathaysian tectonic system. In the east, it is adjacent to Mianduhe fracture and original forest, in the west, in faces the West Wuzhuer Basin with the upper Kuli fault, the southern segment is in the south vicinity of Hailaer regional structure with the Hushan basin and Nantun-Sisumu basin, and in the north, it is adjacent to the Molegeer River uplift.

The terrain gradually decreases from northeast to southwest, with Great Hinggan Mountain for low hills in the eastern segment and undulating high plain in the western proportion. The lowest altitude is 478 m a.s.l and the highest altitude is 700 m a.s.l, 589 m a.s.l on average. The region is of the south of the Mt. Mantou and Mt.Guanjie convergence, which presents a hilly erosion landform subjected to long-term weathering and denudation where mainly features of this sector is generally characterized by flat peak, approximate platform and low topographic slope. The site is distributed in gullies or lowlands between the Hailar River, the Morigler river valley and the wavy high plain, with an aspect of 355° and a terrain gradient of 34° ~ 52°, respectively. According to the surface topography conditions and the overall mine plan, the Baorixile coalfield can be divided into the following gromorphic units: erosion of mountain topography, erosion hills, denudation plains, and accumulation of landform. Since it was put into operation in Baorixile open pit coal mine, landslides of different scales occurred almost every year, which seriously affected the safe production of coal mine and brought.

### Lithology

Field surveys and engineering drilling are typically used to identify the stratum lithology in order to visually display underground information. Borehole investigation disclosed that the stratigraphic succession primarily includes (from old to young) the coal-bearing section of Damoguaihe formation in Lower Cretaceous age, Meiletu formation of Pleistocene Series in Neogene, and few Quaternary formations overlying Neogene sediments.

The uppermost widely distributed layer throughout this region is Quaternary loose deposit with great variation of thickness within the range from 11.70 m to 39.65 m. The main exposed layers are composed of clay with dilute tan, mud-cracked, dense and high plasticity, and a small amount of yellow to yellowish-gray medium sand, fine sand, silt, and a small amount of gravel and red brick humus, and most of them had been removed during surface mining. According to survey data, the aquifer in this area is divided into Quaternary pore aquifer group and Cretaceous fissure pore aquifer group, and the underlying mudstone forms the water-proof floor of the aquifer group. The coal-bearing strata are weakly cemented sandstone, mudstone, and glutenite of the Damoguaihe formation in the Cretaceous age. The buried depth of the coal seam is about 100 m, and the thickness of the coal seam is approximately 23 m. According to the investigation data and the site exposure, 2 ~ 5 layers of tonsteins are intercalated in the coal seam, which are all less than 30 cm in thickness, respectively and nearly horizontally distributed glutenite of Damoguaihe formation (K1d), tuff of Meiletu Formation, and ore bodies. The glutenite (K1d) is widely exposed on the slope surface of the open pit with a thickness of over 100 m, and the color is grey-green or grey white. Its gravel diameter is generally 1-2 cm, showing shape structure with subcircular to subangular. From bottom to top, there are 13 coal seams in total, among them, coal seam *B* is a minable coal seam. The main mineral components of the glutenite (K1d) are carbonaceous mudstone, quartz, siltstone, sericite, and chlorite matter, and weight percentages are 40–75%, 19–38%, 36%, 3%-12%, 47%, respectively. Among them, the carbonaceous mudstone is silky luster and arranged regularly, and the quartz is granular with a particle size of 0.05–0.1 mm. There are also accessory minerals in the glutenite (K1d), such as tourmaline, barite, kaolinized, and perovskite. The glutenite (K1d) is mainly composed of granite gneiss gravel and quartzite gravel with good roundness and easily changed from completely weathered to moderately weathered in the landslide area with the increase to buried depth. This information overlies the glutenite (K1d) with stratigraphic unconformity. The tectonic morphology of this area is basically a fault-depression synclinal basin with near east–west trend and controlled by basin-margin faults respectively. The open-pit ore field is located in the central part of the basin, and the inner layer of the ore field is gentle. The overall tendency is west or southwest, and the dip angle is generally about 5°, with local microwave fluctuation. There are 8 faults in the ore field, including 7 normal faults and 1 reverse fault. Faults are mainly concentrated in the north and west of the mining boundary, and the mining will be affected to some extent when the mining engineering develops near the fault. The southern and central coal seams of the mining boundary are almost free of fault influence.

### Hydrogeological and rainfall condition

The survey region appertains to the continental subtropical monsoon climate, with an annual average temperature of – 2.4 °C. The lack of precipitation limits the recharge of aquifers. The concentration of rainfall and the long time of snow cover determine that the recharge period of precipitation is mainly concentrated in June, July, August and September (rainy season) and snow melting period (late April to May). The highest extreme temperature was 38.4 °C in August with an average of 21 °C, and lowest extreme temperature was -49 °C in January with an average of -23 °C. In the site, the annual rainfall is about 322.2 mm and the maximum daily rainfall is 5 mm. There are few days of rainstorms and most days of light rain. Evaporation in the area is higher in July and August, and the average evaporation is 1058.5 mm.

In this region, the occurrence of groundwater principally origins from atmospheric dewatering infiltration, coal mine goaf sources, and Quaternary pore water. To the extent, the flow in this environment has also been described by Xia et al.^46^. As groundwater behavior is governed by the conductivity and roughness of cracks and tiny fissures. Discharge of groundwater occurred at surrounding springs and rock cuts. Additionally, Morgler River, running through the east basin from BaoRixile open pit coal mine, likewise converges the groundwater from the coal mine. Groundwater outcropping points are occasionally surveyed perimeter the open pit in the process of field detecting.

**Fig. 7:**
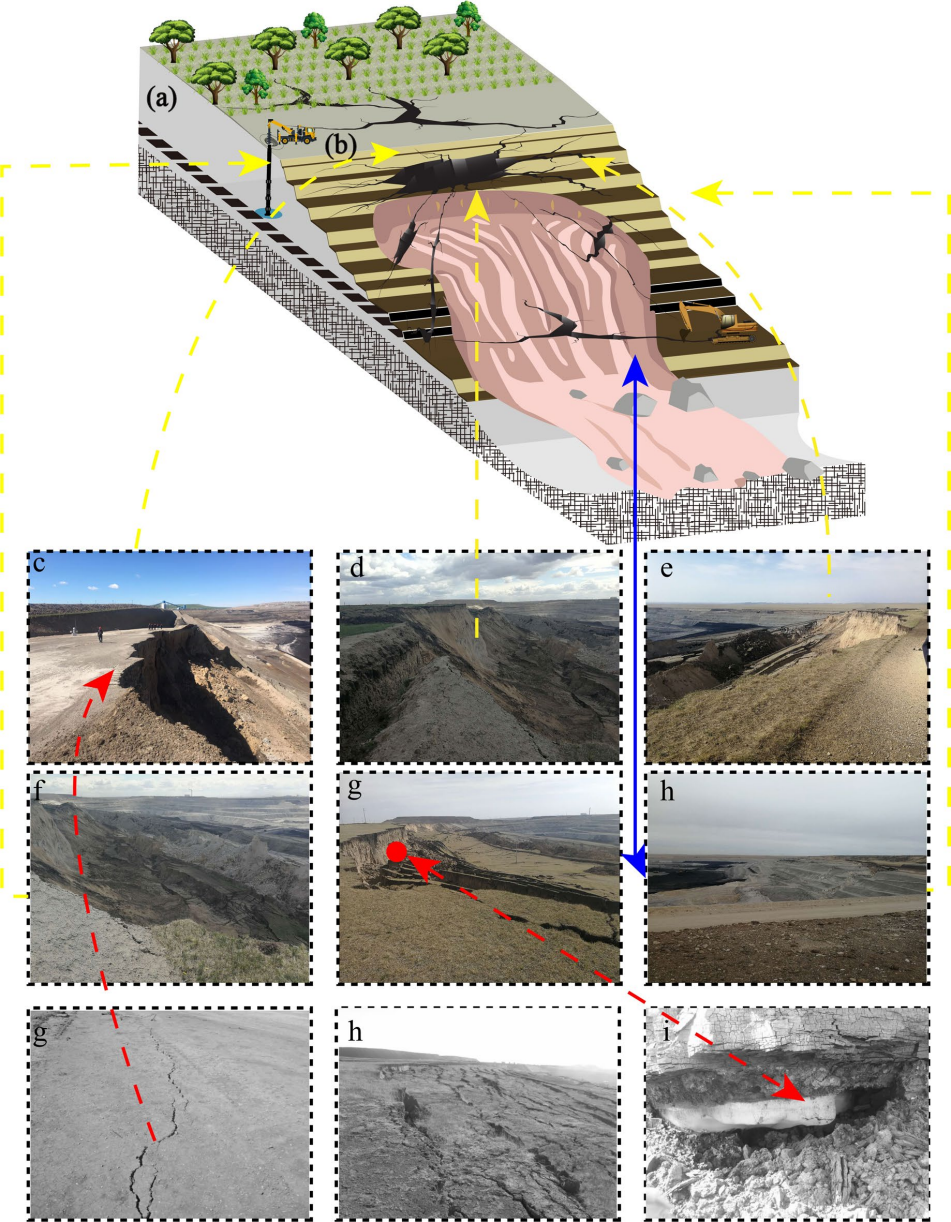
Distribution of (**a**) Annual precipitation (2005 ~ 2020); (**b**) Annual precipitation and evaporation (2011 ~ 2020); (**c**) Daily precipitation and Cumulative precipitation (2019 ~ 2020) in the study area.

Figure 7 displays the rainfall data of monitoring station No. 85689 which is from the landslide site. It can be seen that the rainfall was fairly intermittent in April 2020, there was at least 1 month of rainfall exceeding 10 mm. Noteworthily, precipitation capacity is strong during this period and the rainfall reached 15.3 mm and 14.3 mm on April 13 and April 23, respectively. This indicates a substantial daily rainfall of 38.3 mm on 29 April 2020 at the site. Landslides and debris flow oftentimes transpiring on the account of a conjunction of plurality quantity of accumulated rainfall and great rainfall intensities^47–49^. Consequently, the cumulative rainfall pattern was conducive to the landslide. We generalized the incidence of rainfall on slope failure on two aspects. On the one hand, failure event predominantly occurs due to rainfall infiltration increasing pore water pressures within the slope, which reduces effective stress in turn decreasing the shear strength of rock mass. On the other hand, the strength and stiffness of rock mass will decrease via a whole range of mechanical and hydrological induced weakening effects. To make affair worse, no drainage networks and related actions such as gutters, drainage, and catchment-pits were constructed on workbenches or behind the crest to runoff and obstruct the water. These circumstances could yield advantageous conditions for slope failure occurrence. We summed up that continuous heavy rainfall during April 2020 made the slope vulnerability and is one of the main factors that triggered the landslide event.

### Equipment, datasets and methodology

**Fig. 8:**
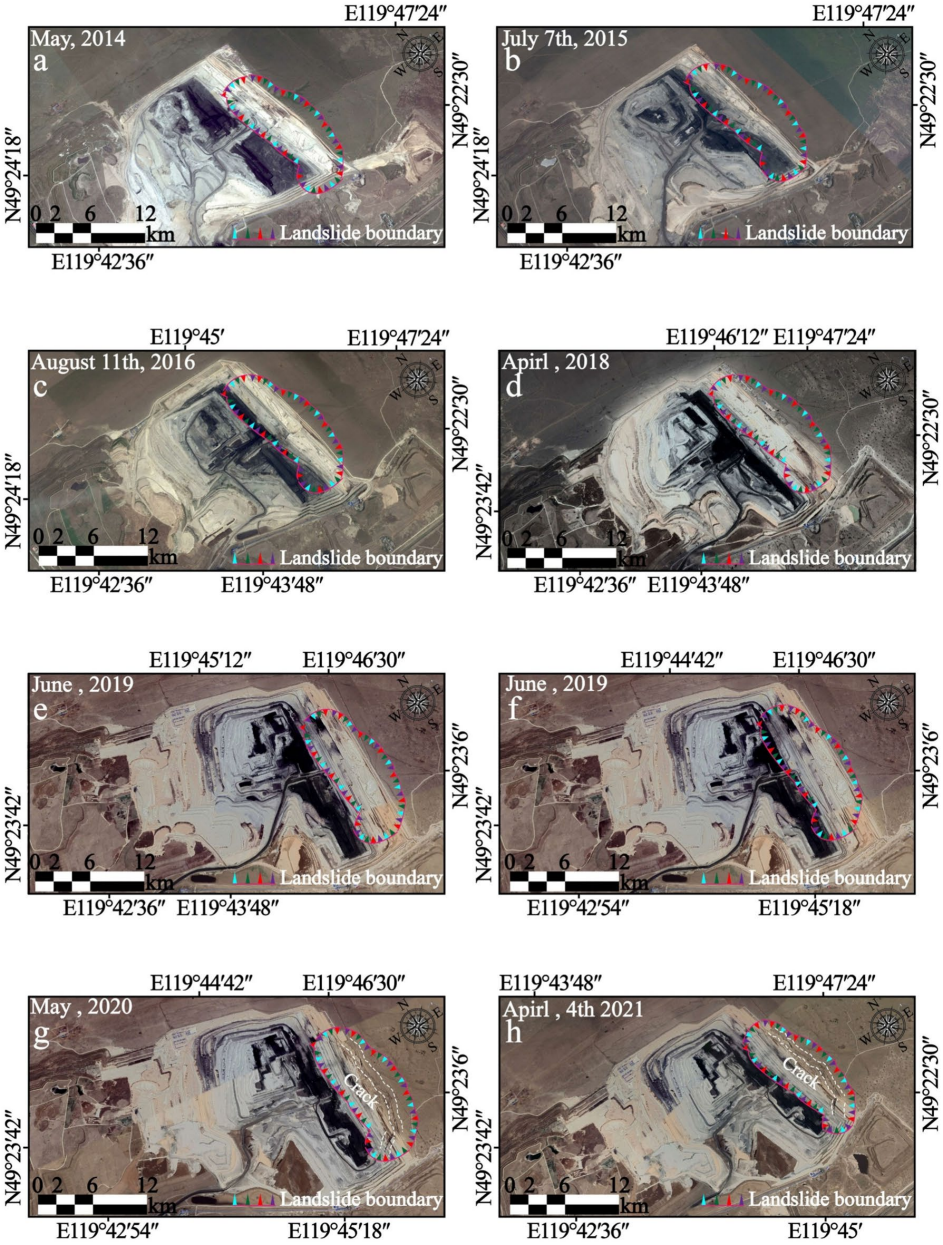
Methodology flow chart of surface measure techniques, UAV campaign, and digital georeferencing.

**Fig. 9:**
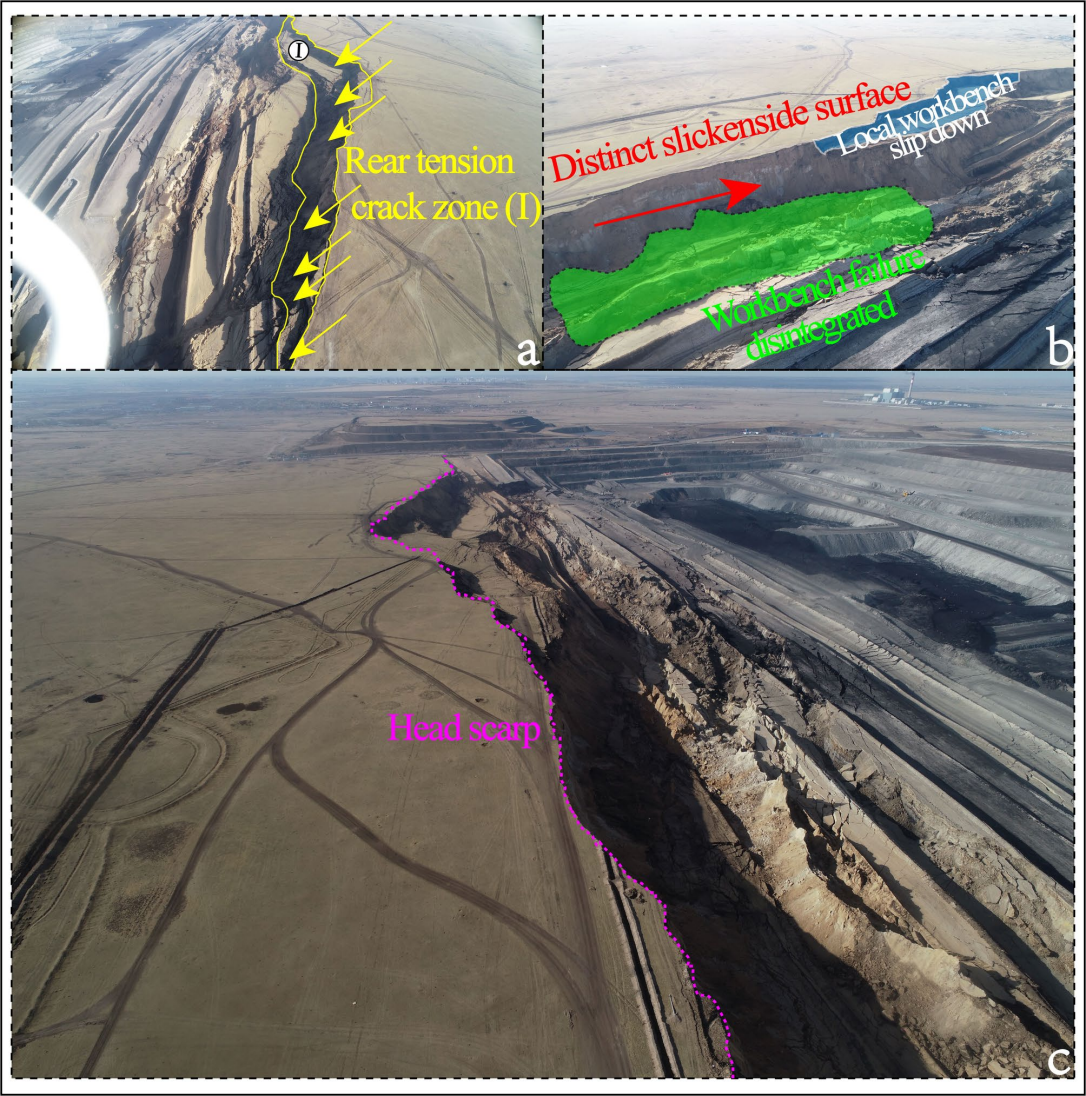
Gray scale of different frames.

An integrated monitoring system was designed and established during the open-pit mining activities. All the equipment used in the current study is presented in Fig. 8 and analytically presented in the following subsections. The monitoring system consists of geodetic global navigation satellite system (GNSS), GPS, inclinometers, automatic crack monitoring and artificial crack monitoring. By processing and interpreting these data, we can obtain historical geoenvironmental conditions, deformation variations and kinematic features of landslides. Every frame image of the landslide process is captured and processed (as shown in Fig. 9). The main flowchart is shown in Fig. 8.

### Unmanned aerial vehicles

The flight campaigns over the landslide area were performed in different dates, the multi-rotor aircraft with the off-the-shelf and custom-made Feima (D200, produced by China Shenzhen Feima Robotics Co., Ltd) UAV. The working pulse repetition frequency, maximum measuring range, and maximum scanning speed of the scanner were 100 kHz, 250 m, and 100 scans per second, respectively. It weighs less than 6.5 kg and can fly for up to 48 min using four-cell 626wah Li-Ion polymer batteries. The custom-made UAV that was used for this study (the flowchart is shown in the center part of Fig. 8) is a hexacopter that bears two camera gimbals. It has a stabilizer in order to absorb all the distortions. The gimbals allow the simultaneous capture of vertical scenes and oblique scenes to realize different aspects of the same area. The camera that the gimbals bear is one Sony A6000 multi-color Edition action camera. The RGB Sony camera is capable of capturing photographs at 24 MP (3000 × 8000 pixels), have a focal length of 20 mm and their pixel pitch is 1.55 μm. In addition to the gimbals, the UAV is powered by two batteries that offer a total flight time of about 15 min. Having this flight autonomy, the specific UAV can cover areas of 0.16 km^2^ in a single pass. Its positioning accuracy is 2.5 m horizontally and 0.8 m vertically.

### GNSS measurement position solutions

**Fig. 10:**
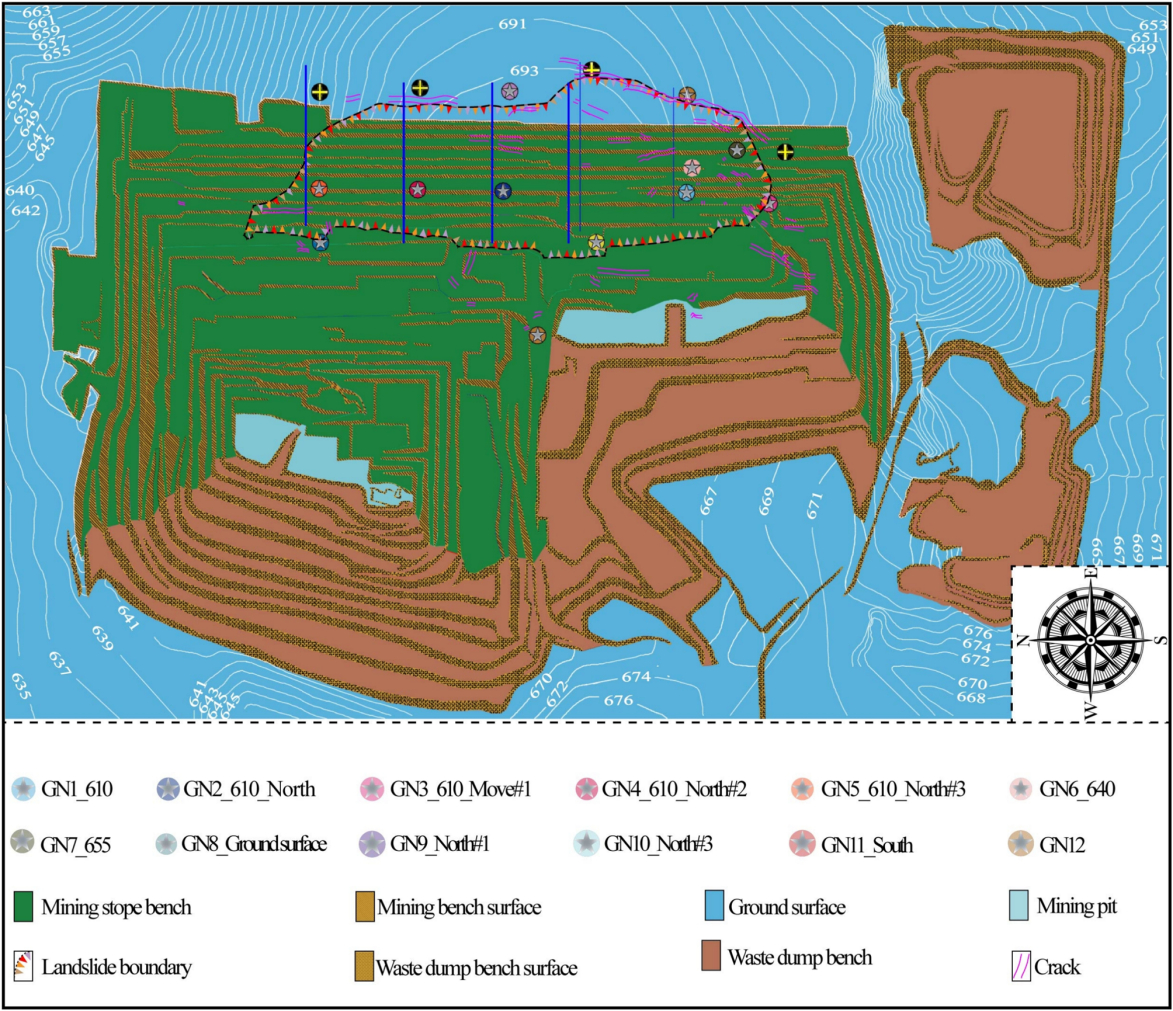
Cracks and fissure monitoring points distribution (Noted, Generated by the SMcad software V7.0 independently developed by Liaoning Technical University).

Since 2004, the south side and west side of the eastern slope in the second mining area have successively dislocated along the weak interlayer in the coal seam, with a dislocated distance of nearly 50 cm, forcing the coal mine to abandon the coal wall at the foot of the slope, resulting in resource waste and rising production costs, disturbing the mining sequence and causing huge economic losses. As the east slope is widely distributed and involves many mineral resources, a set of comprehensive monitoring method is established to grasp the deformation and dynamic characteristics of the east slope. According to the specific geological environment of the eastern slope, a comprehensive monitoring system based on crack monitoring and real-time GPS, supplemented by other monitoring means is determined. On the premise that the installation of monitoring points and the convenience of monitoring are satisfied, four monitoring profiles (Fig. 10) almost coincide with the exploration profile are arranged in the study area, and the key positions to control the deformation and failure of the landslide are selected according to the previous deformation positions of the landslide. The principle of GNSS measurement in the study area was performed according to the pull-wire displacement sensor with the accuracy of 0.1 mm and GNSS points were configured on the stable rock outcrop of the workbench with the elevation of 610 m, 625 m. 640 m, 655 m, and 685 m, respectively (Fig. 10). The naming principle is “point number + elevation + monitoring mode (including monitoring location)”. For example, “GN2_610_North” means the monitoring point at elevation 610 on the No.2 monitoring profile. In the GNSS measurements, 5 monitoring the baselines for GNSS receiver sets, to be two GR30 and one ME7 measurement engine affiliated to Tsinghua University Department of Hydraulic Engineering, and two Ashtech Z-Max and one Satlab SL600 GNSS receivers belonging to Huace enterprise, were used since November 2005. For the GNSS points within the east site, at least One-hour interval measurement for each was performed in a persistent way. Moreover, we have adopted the double-difference static positioning mode of RTKLib to estimate the position of all the Baorixile landslide GNSS sites. This approach desires to define a reference benchmark station whose measurement epoch coordinate values are during the estimation procedure. We have proposed one processing in order to estimate how the distance from the reference benchmark station and the length of observation can influence the estimation of the position and the corresponding values.

### Artificial crack monitoring

With the intention of more sufficiently collecting pertinent information such as the cracks depth, the run-through conditions, tiny sensitivity detecting, and interconnected evolution process between shallow ones and deeply penetrating ones, some other monitoring and datum sampling methods used in the study are listed as follows:

- Twenty eight in-place inclinometers (Model KXO-1, produced by Changji, Incorporated). The horizontal deformation was measured automatically and continuously.
- In comparison with the automatic GNSS monitoring, although it is real-time monitoring and its data can be transmitted, a self-manual could get a high sampling frequency.

### Augmented digital image correlation (AL-DIC) method

Digital image correlation (DIC) is a prevalent technique for measuring full-field 2D surface displacement to different datasets to test the potentialities and limitations. This digital approach can be used to analyze and manipulate attainable imagery database, and miscellaneous categories of information can be extracted relying on the typology of the selected image procedure technique. Deformations are computed by dint of contrasting and processing of coregistered digital images of the surface of the identical “object” collected the undeformed and the deformed event. In most cases, the DIC technique allows for displacements/deformations to be measured without the installation of sensors/reflectors in the measured object; i.e., it can be regarded as a fully remote measurement system. Notwithstanding, the fundamental prerequisite for DIC analyses is the occurrence of a random speckle pattern on the object surface, which is crucial for acquiring an exclusive explanation in the correlation process. Essentially, identifying the correspondence between single pixels in two images is impossible because the intensity value of a single pixel can typically be found in thousands of other pixels in the post-event image. Consequently, unique correspondence does not occur. Therefore, consistency between two speckle patterns is accomplished by considering a pixel and its neighborhood in the pre-event image (ƒ) and searching for the same subset in the post-event image (g) (the flowchart is shown in the right part of Fig. 8). Hence, the correlation processes have to refer to a region of interest (ROI). The ROI represents an area of the picture chosen by the operator, and it is overlaid only on the object to be correlated. Therefore, between the reference and the deformed images, the ROI represents the analysis mask in which the correlation algorithm operates.

**Fig. 11:**
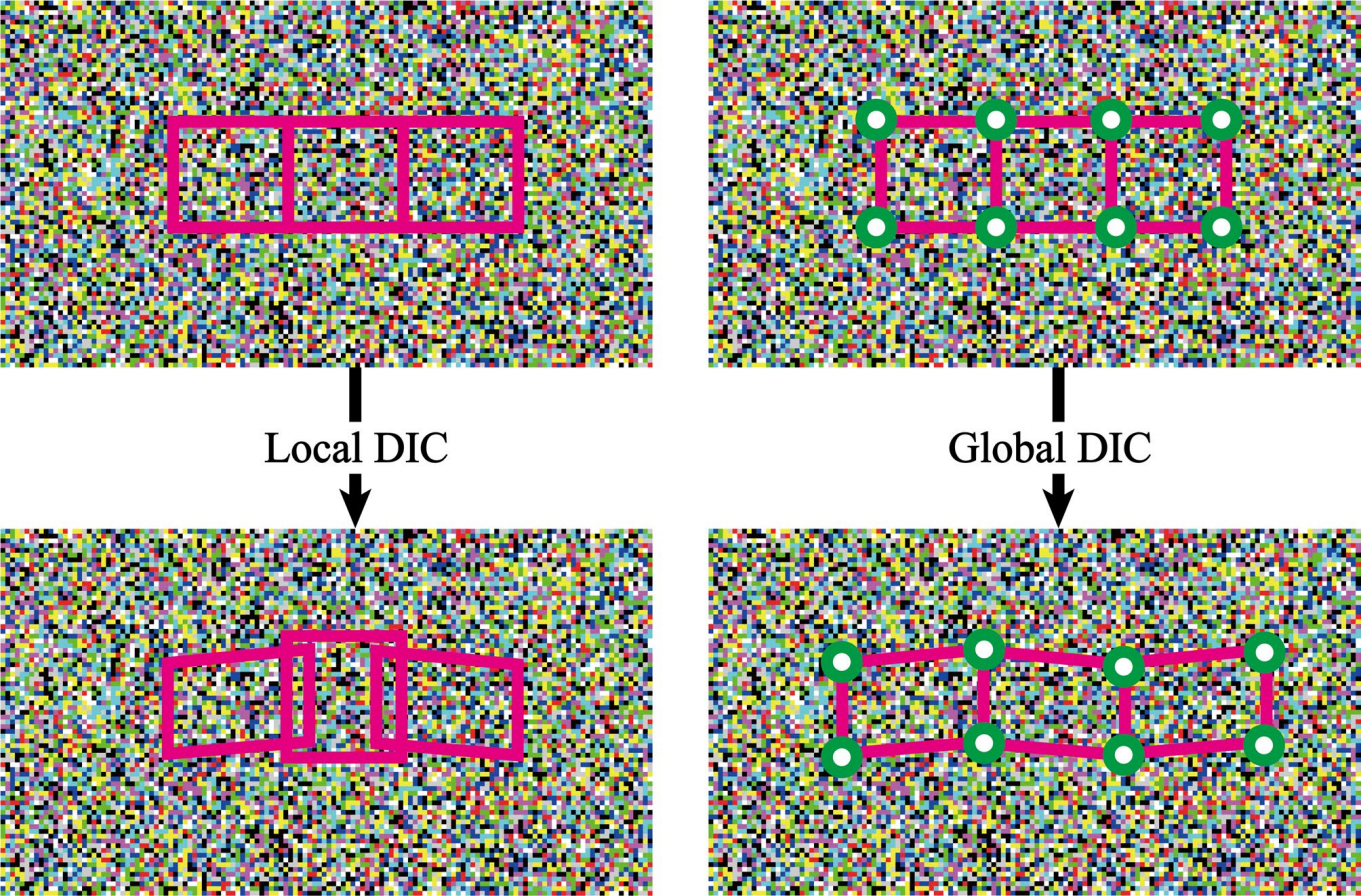
Comparision between Local and global DIC method.

Over the last thirty years, various DIC algorithms to compare images and to obtain displacement and strain have been proposed and implemented. Most algorithms can be cast into two categories: local subset DIC method and global DIC method. We apply a new image comparison algorithm, Augmented Lagrangian DIC (AL-DIC), which seeks to combine the advantages of both the local subset DIC (speed and parallel implantation) and the global DIC (displacement compatibility and strain smoothness). Mathematically, the rationale of Local Subset DIC breaks up ROI domain into a finite number of subsets, and make the ansatz that the deformation is piecewise affine (see Fig. 11, left)

1 $$\Omega = \bigcup\nolimits_{i} {\Omega_{i} }$$

2 $$y(X) = X + \sum\limits_{i} {(u_{i} + F_{i} (X - X_{i0} ))} \chi_{i} (X)$$

where $X_{i0}$ is the center of local subset $\Omega_{i}$, $u_{i}$ is the displacement of $X_{i0}$ and $F_{i}$ is the uniform displacement gradient of $\Omega_{i}$ and $\chi_{i}$ is the characteristics function.

In the global DIC method, the global deformation uses a global basis set based on a finite element discretization, such that the compatibility or continuity of displacement is guaranteed automatically (see Fig. 11**,** right)

3 $$y(X) = X + \sum\limits_{p} {u_{p} } \psi_{p} (X)$$

where $\psi_{p} (X)$ are chosen global basis functions and $u_{p}$ are the unknown degrees of freedom.

Recall the ansatz in Eq. (2) we make in local subset DIC. In this ansatz, the local displacement $u_{i}$ and local displacement gradient $F_{i}$ in the subdomain $\Omega_{i}$ are independent of each other, and independent for each *i*. Thus, there is no guarantee of compatibility for deformation. However, if the displacement field were compatible, then the displacements and the displacement gradients would not be independent, but instead satisfy a global constraint

4 $$\left\{ F \right\} = D\left\{ u \right\}$$

where D is the discrete gradient operator that depends on the discrete gradient operator that depends on the discretization. The local subset DIC ignores this constraint while the global DIC enforces this constraint by kinematic construction.

The key idea of AL-DIC is to treat this constraint (4) efficiently. We do so using an augmented Lagrangian method. Specifically, we solve this problem using an alternating direction method of multipliers that allows us to break it up into simpler problem. A balcony at the edge of the landslide area housed a digital camera. The digital camera exactly recorded the entire process of the slope from obvious hints of surface fracture to eventual instability, which lasted for 2′34″ with 30 frames per second (FPS). As shown in Fig. 9, slope images at 8 moments are selected to present the images of slope impending failure for digital image correlation analysis.

To consistently segment the failure process, we derived three kinematic invariants from the AL-DIC displacement field u(x,y); (i) the cumulative displacement magnitude $U = \sqrt {\mu_{x}^{2} + \mu_{y}^{2} }$, (ii) the maximum in-plane principal strain ${\varepsilon }_{1}$ from the symmetric part of $\nabla \mu$, and (iii) the in-plane shear strain ${\gamma }_{xy}$ We then defined four data-driven stages using percentile-based thresholds and spatial-coherence criteria within the ROI.Stage I (pre-failure creep): U and ${\varepsilon }_{1}$ remain below the 75th percentile; no persistent high-strain clusters.Stage II (crack initiation & bench warping): emergence of contiguous (> A pixels) clusters where ${\varepsilon }_{1}$ or ${\gamma }_{xy}$ exceeds the 90th percentile; intermittent uplift–subsidence patterns on local work benches. Stage III (localized sliding–subsidence): rapid growth and coalescence of high-strain clusters (exceeding the 95th percentile) with directional consistency in U. Stage IV (run-out/toppling): widespread high-strain footprint with sustained displacement gradients directed towards the anterior colluvium zone. All thresholds were validated by sensitivity checks (± 5 percentile points) and cross-checked with field crack-monitoring logs and UAV videos to avoid single-metric bias. The ROI and reference/deformed pairs follow the AL-DIC workflow described above.

We combined four complementary data streams to capture the landslide’s conditioning structures, 3-D geometry, and time-resolved kinematics. First, detailed geologic/geomorphic mapping constrained material units, weak interlayers, bench layouts, and deformation zonation (rear tension cracking, top detachment, middle sliding–subsidence, anterior colluvium), thereby defining the ROI and guiding sensor placement. Second, UAV SFM-MVS photogrammetry provided co-registered ortho-mosaics and DEMs before/after the event to quantify elevation and volume changes and bench warping; these products supplied geometric control and stable features for co-registration with field observations and video frames. Third, GNSS and crack/tilt monitoring delivered long-term displacement time series that anchored AL-DIC staging thresholds (from creep to acceleration). Finally, AL-DIC on the 30-FPS video yielded full-field displacement and strain maps and a four-stage hazard chain. Spatial concurrence between AL-DIC high-strain clusters and mapped cracking/bench tilting, temporal concurrence between AL-DIC stage transitions and GNSS/crack accelerations, and metric consistency between AL-DIC displacement gradients and UAV-derived elevation/volume change collectively cross-validated the results and strengthened causal inference linking rainfall/freeze–thaw, weak interlayers, and macro-scale slide–toppling.

Utilising the aforementioned combined approaches, we can precisely identify the various stages of the landslide and ascertain its triggering reasons and inherent geological conditions. Subsequently, in the findings section, we will demonstrate how these methodologies efficiently elucidate the instability process of the landslide and its principal triggering causes.

## Results

### Deformation and destruction characteristics

In accordance with the field geological survey and geomorphologic expressions, the landslide area can be divided into four subunits zones in terms of geomorphology, deformation characteristics, and material structure: the rear tension cracking area (*I*), the detachment zone at the top (*II*), the middle sliding subsidence area (*III*), and the front colluvium area (*IV*) (Fig. 5a–c).

### The rear tension crack zone (I)

This portion was located at the crest of the entire deformation zone with the elevation of 700 m a.s.l. to 685 m a.s.l., (Fig. 5b) the height difference is 30.8 m, the distance across the northern and southern lateral flank was approximately 1720 m, the ballpark estimated volume is up to 6.5 × 10^5^ m^3^. The failure pattern of the rear tension crack zone exhibited traction deformation and demonstrated the occurrence of notable downhill displacements. The survey revealed that considerable amounts of new-formed fissures, fresh scarps and arc opening cracks have been found on the road of the landslide surface.

**Fig. 12:**
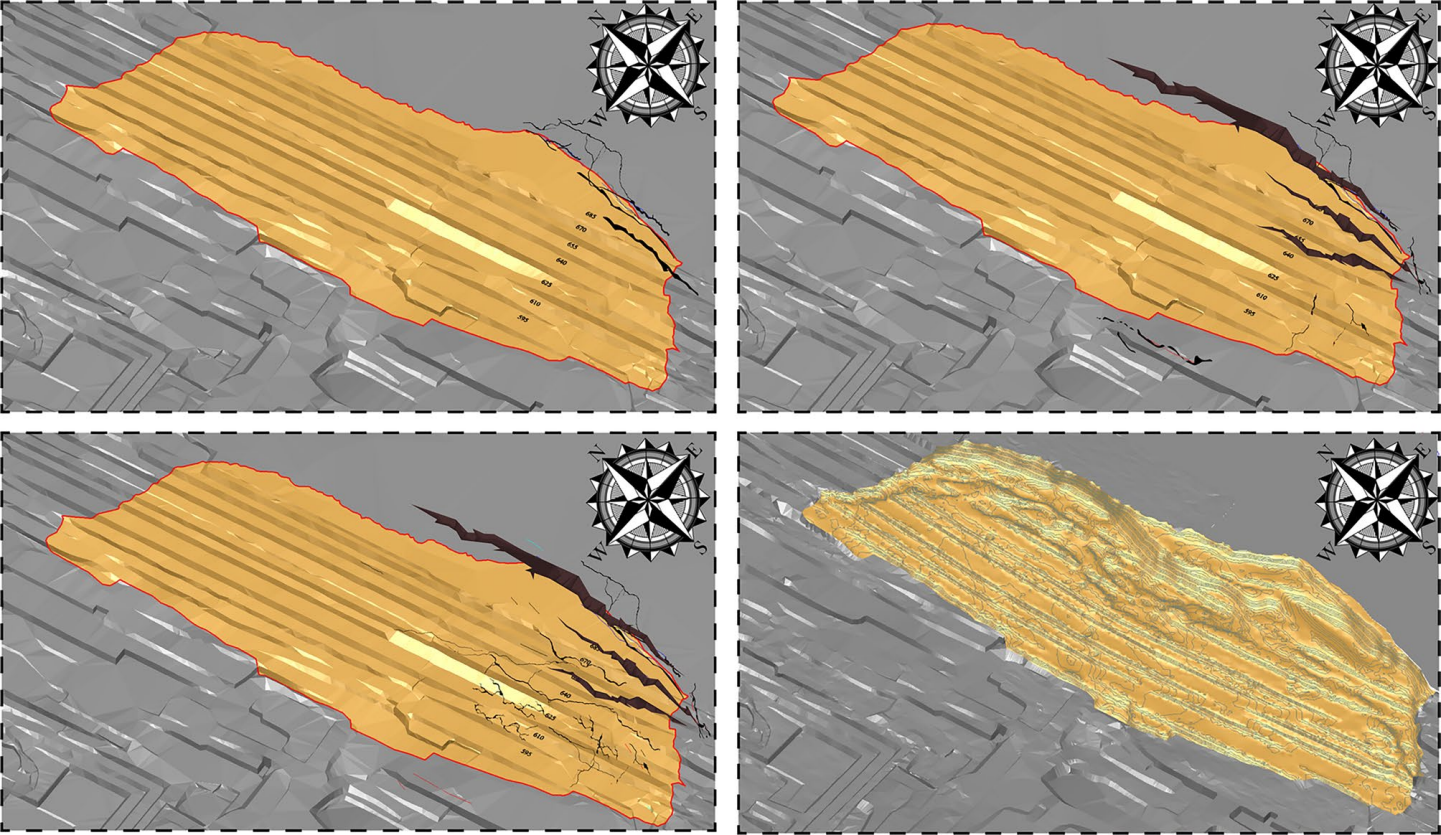
Series of historical orthoimages of Bao Rixile open-pit mine from 2014 to 2021. (Noted, Fig. 12 is made from the cross-platform map browsing software developed by Interactive Map Software Co., Ltd.).

**Fig. 13:**
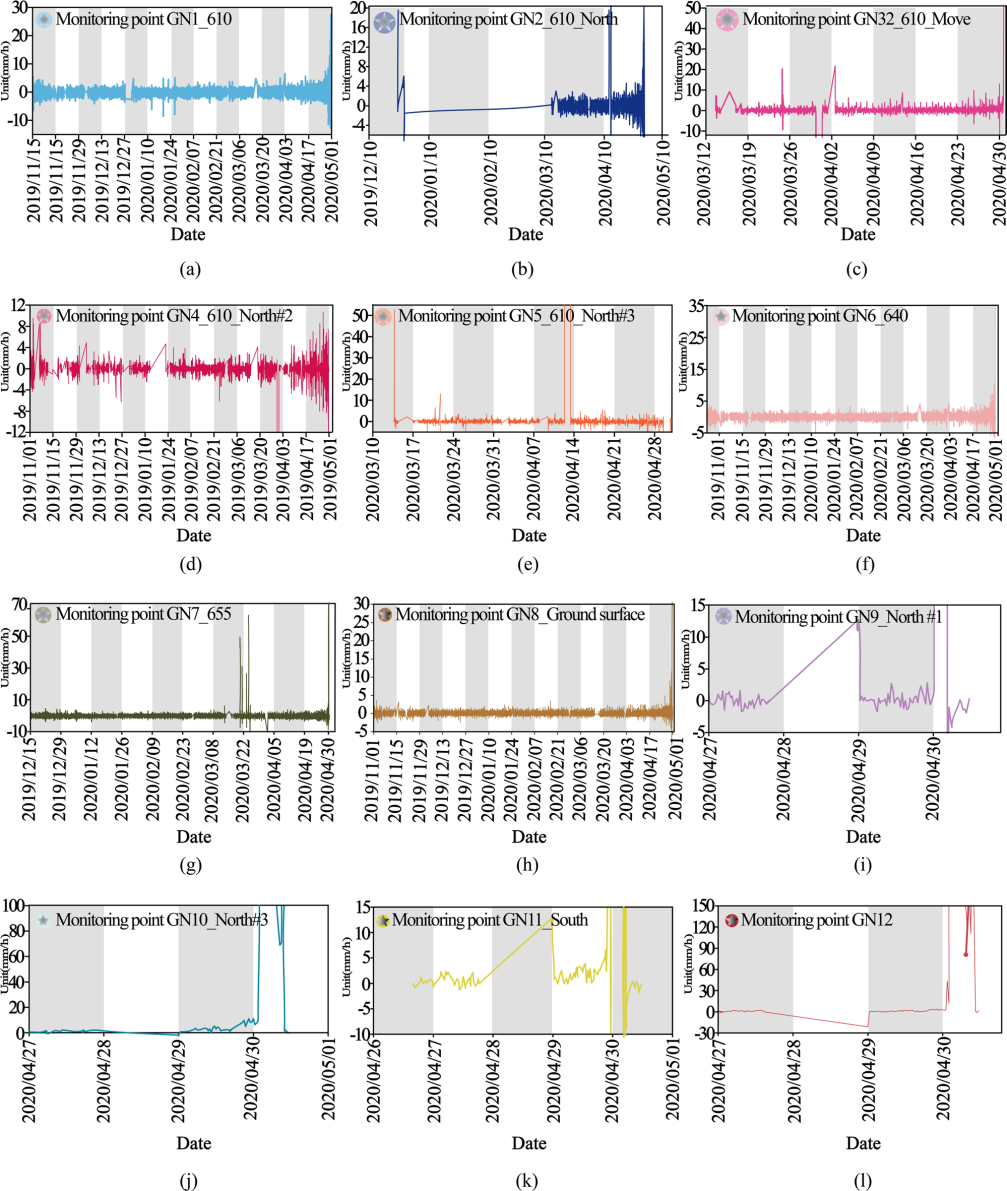
Processing of landslide evolution of landslide (Noted, Generated by the SMcad software V7.0 independently developed by Liaoning Technical University).

**Fig. 14:**
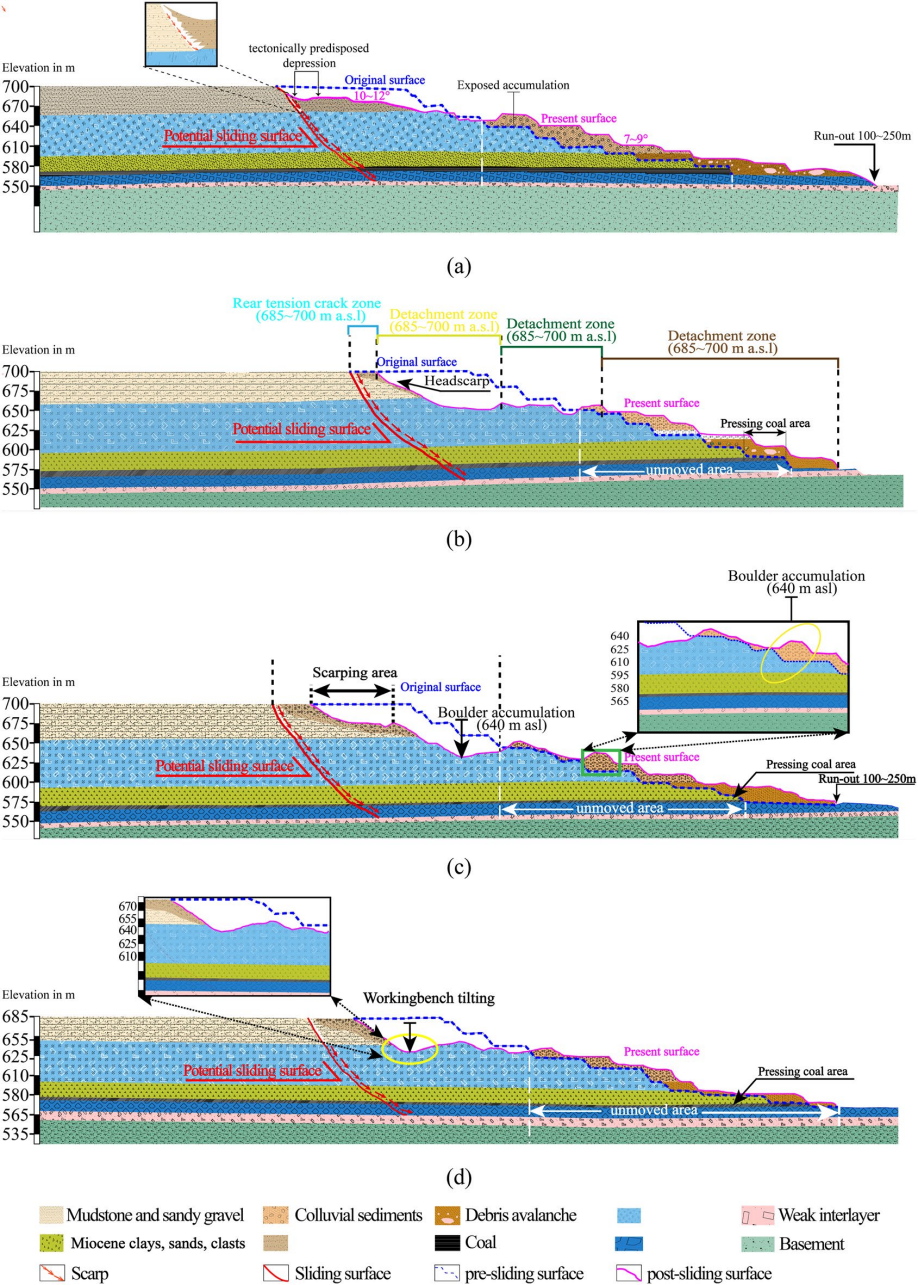
Crack monitoring results of a series distribution monitoring points.

Numerous minuscule fissures and surface tension cracks, measured to be with lengths of approximately 5 to 8 m, typically 1 to 40 cm in widths, and visible depths of 10 to 50 cm thereabouts, developed widespread around the crest of the slope and nearly parallel to exposed faces of slopes. To follow the crucial movements before the landslide, eight remote sensing images of Google Earth with 0.6 m precision from 2014, 2015, 2016, 2018, 2019, 2020, and 2021 are examined (Fig. 12a–h). The verbal instruction secured from local dwellers demonstrates that the landslide has been active for at least 5 to 6 years duration, which has concordance with remote sensing images from Google Earth (Fig. 12). From May 2014 to July 2015, no apparent could be encountered in the Google Earth images in 2014 and 2015, insinuating that the movement of the landslide body from 2014 to 2015 might be rather feeble (Fig. 12a,b). From August 2016, the circumstances of the southeastern portion of the landslide aggravated (Fig. 12c–f), and plentiful deformation (for instance, faint subsidence, minor tension cracks, and localized collapse could be discerned on the landslide surface. Particularized site investigations and enquiring vicinity shepherd and scene operators disclosed the cracks expanded and winded progressively to the north at the rear locality of the landslide since January, 2020 (Fig. 12g,h). In the meanwhile, the deformation characteristics and evolution processing of the landslide area are synchronously are analyzed and explained combining to the surface profile feedback from the digital elevation model (Fig. 13) obtained pre- and post the failure event and the crack monitoring datum (Fig. 14a–l). In the time domain from November 2019 to the end of March 2020, although the crack monitoring data fluctuates as a whole, it is due to the mining effect caused by coal production and the blasting vibration caused by excavation. Under the control of the safety range of the open pit mine slope, it is enough to maintain the safety of slope body. Monitoring point GN2_610_North, GN3_610_Move, GN5_610_North#3, GN7_655, and GN9_North#1 showed a jump rise in early April 2020, respectively. These points are concentrated southeast of the landslide area, as well as sporadically scattered in the trailing edge of the research area. The peak value varies from 15 mm/h to 70 mm/h. Among them, point GN7_655 jumps most obviously, and the actual change image is also consistent with the indications of this monitoring site. It is preliminarily concluded that the landslide event started from the trailing edge.

### The detachment zone at the top (II)

This deformation zone is roughly between the 685 m a.s.l and 655 m a.s.l. contour lines defined by the mine engineers through field and UAV surveys carried out in the aftermath of the event. Numerous bluffs and rock collapses at the back-scarp of landslide show the destruction pattern attributes to the local vertical toppling failure. Based on the report, this deformation zone of the pit had a depth of about 30 m, which consisted mainly of two 12-m benches and one 6-m semi-bench on the uppermost three platforms. The main scarp of the post-failure residual bluffs consisted principally of completely weathered phyllite (Lph) and surface clays (Fig. 6c). The lithology strata in the elevation of this deformation zone are virtually parallel to the workbench of the open pit and then have a tabular blocky structure. It can be seen from (Fig. 6a,b) that the original rock structure had been disturbed and displaced. Due to the action of gravity slide-pushing force and compressive stress *σ*_p_ and shear stress *τ*, the back-scarp are rolling downwards into the mountainside, indicating that the sliding surface developed along the metamorphic joints. According to characteristics (profile *I*-*I*, Fig. 5a and b) of its offset the fractures can be fourthly segmented into 2 subunits: (a) tension-shearing fractures-found on the upper part of the slope in this formation zone (Fig. 14a and b), showing significant downhill displacements; (b) bench tilting, the excavated bench tilts over, causing damage of slope.

### The middle sliding subsidence zone (III)

Deformation zone ***III*** represents the central part of the slide, which is evidenced by distinct, alternating uplift and subsidence zones within localized workbenches. This area is approximately 1780 m long and 480 m wide, covering an area of X × 10^4^ m^2^. The back-scarp in this deformation zone has been taken as a evident boundary to separate zone *III* and zone *II*. The elevation of the front is approximately 635 m and that of the trailing edge is 137.8 m. In the elevation range of 655 to 635 m, the platforms and workbenches of this deformation zone are principally impacted by uplift, whereas uplift occurs atthe higher elevation in this zone. In the elevation range of 635 to 610 m, the lower zone of slide middle segments was affected by subsidence (grain-flows and rockfalls). The on-site prospecting and surveying of post-failure disclosed the subsidence zone has miscellaneous sizes of rubbles, particulates and blocks. The sliding masses are consequently disintegrated much more rapidly when it was sliding. Some large rocks uplift and roll down on the underlying the relatively small diameter rocks. Surficial materials also encompasses many intact large block stones with a average diameter of 2 to 5 m with maximum width of 128 m. Due to its huge gravitational effects and the squeeze effect lateral high speed landslides, the rock block slide possessed is relatively low-velocity but high-momentum.

### The anterior sliding colluvium zone (IV)

As shown in Fig. 5d, the failure signs of potential shear outlets at the anterior edge exposed on the convex flank with irregular bead-like shaped under the influencing seasonal outflowing water, strong weathering, and muddy rocks were observed (Fig. 4b). This deformation zone was situated at the anterior section of the whole deformation area. The elevation was between 550 and 580 m a.s.l, and the colluvium mass was constitutive of the collapse of the weathered rock and surface clays.

### Analysis of AL-DIC simulation results

In this method, displacement image entropy is used to measure and statistics the chaotic degree of slope change information in this modified method. Therefore, image information entropy is used as a metric standard to measure the abundances of information in different areas of slope image. The greater the information content of the image, the greater the randomness of the image, and the better the correlation matching effect is when the improved digital image correlation method is used for calculation.

**Fig. 15:**
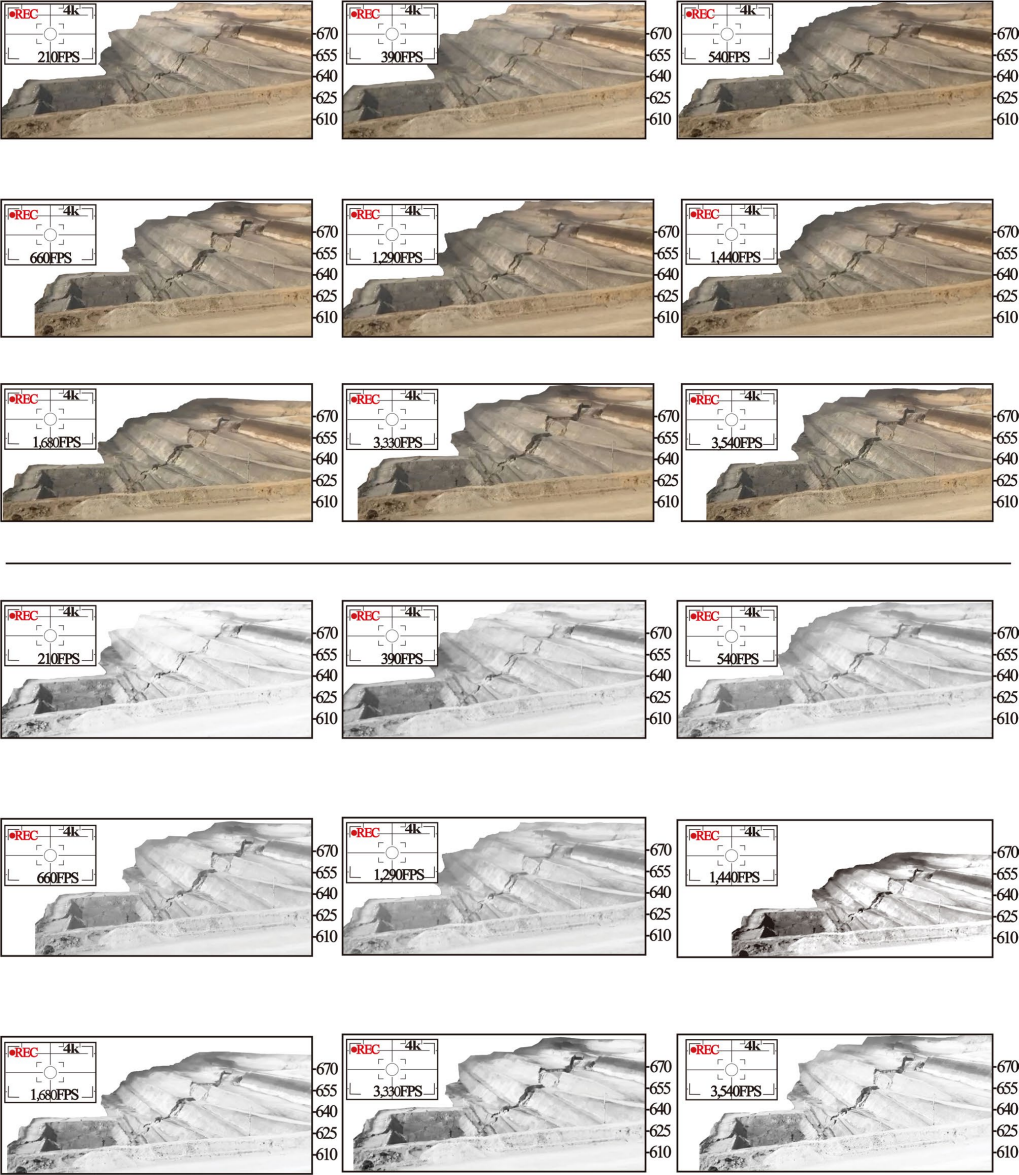
X direction displacement and image information entropy.

**Fig. 16:**
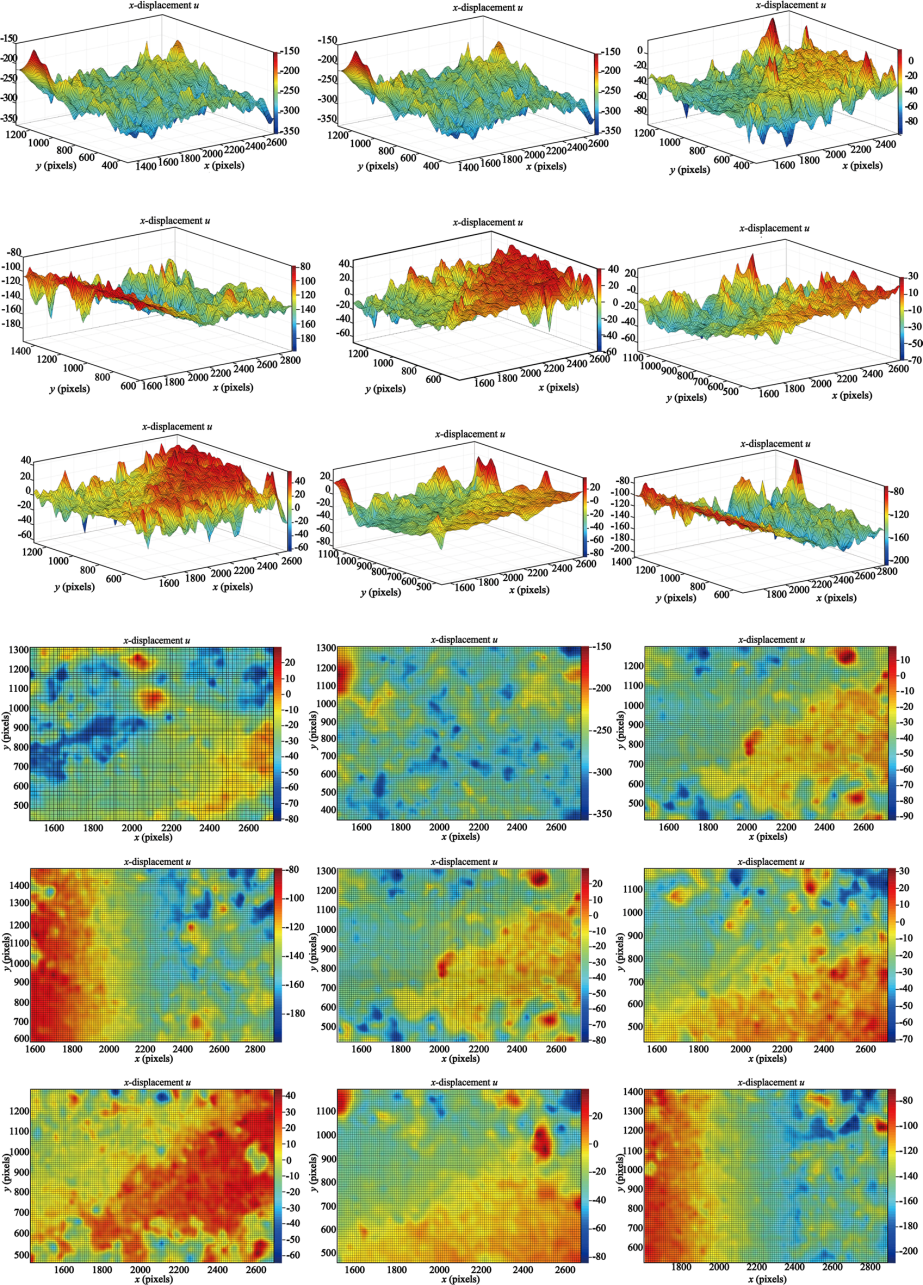
Y direction displacement and image information entropy.

The gray images of different frames were extracted and the information entropy of the images in the process of slope deformation in *X* (Fig. 15) and *Y* (Fig. 16) direction were calculated. In the process of frame number 210 ~ 390 FPS, the entropy value of the information entropy is low, and the local shape variable of the 2d deformation cloud mao has an increasing trend, indicating that the landslide is in the initial starting stage, but it does not have enough identification. At the FPS 540, 660 and 1290 FPS of the information entropy measurement proportion in Figs. 15 and 16, the displacement deformation value fluctuates sharply and the variation characteristics are obvious. Combined with real working conditions, the steps in the landslide area are mainly traction motion and slope step tensile crack, and the vertical collapse motion is weak, so the information of image displacement change in this stage is abundant. At this stage, image analysis can be better reflect the real state of slope movement.

It can be seen from the calculation results of Al-DIC analysis (Figs. 15 and 16) that the displacement deformation diagram of 9 frames in the *X* and *Y* directions is basically consistent with the variation process of traditional landslide displacement evolution curve over time: initial deformation, constant velocity deformation and accelerated deformation. In the initial deformation stage, the slope body just begins to deform and the variation is small. In the constant deformation stage, the deformation curve shows a linear slow growth, which is characterized by the increase of the failure area in the image analysis results. At 1680FPS, the deformation increases rapidly until the slope failure occurs.

The aforementioned data indicate that the interplay between extreme climatic circumstances and mining operations is crucial in the incidence of landslides. Subsequently, we will examine the ramifications of these findings for landslide prediction and prevention tactics in mining regions, and deliberate on how to use these insights to enhance future safety management protocols in such places.

### Landslide mechanism and trigger factors

#### Landslide mechanism

Initially, season seriously rainfall is the main factor to trigger landslide to slope stability. Although there is no phreatic water distribution in the stope, the weak interlayer has a high content of clay, which is attributed to good impervious bed. When meteoric precipitation infiltrates through stratigraphic layer, it converges at the weak interlayer, which leads to the increase of water content in the weak interlayer and the degrading of strength and effective stress, etc., thus causing the slope instability.

After the disaster, kinematic analysis was performed to identify the type of the instability to discover the landslide mechanism. On the site slope near the rear boundary of the landslide, the local failure had the appearance of a tremendous plow wedge, carrying away the rocks and the debris on the slope. The progressive failure mechanisms happened in this section can be attributed in two aspects: (i) The complex geological structure and well-developed rock joints and fissures in the mining area create conditions for the extension and slip of rock mass; and (ii) surface excavation activities for construction of transportation roads are inclined to reduce the stability of the slope as in situ stresses *σ* are lower due to the dropping effect of gravity. Additionally, the failure characteristic of the rear tension crack zone showed tractive sliding and indicated the occurrence of significant downhill displacements. Tangential stress *σ*_t_ was altered due to multi-stage mining benches and engineering slopes. With the continuous exploitation and stripping work, one certain layer under work benches was cut off in the process of mining pit deepening and widening, which caused the rock mass in the free-face state and destroyed the natural state of the original rock layer. When the shear strength of the weak layer in the slope is not enough to resist the sliding force generated by the overlying body weight, slope deformation occurred and rock mass slided westward. The stress of rock mass in this section is “active”, which corresponds to the “active stress zone” in mechanical mechanism. In order to balance the sliding force of the middle and upper rock masses, the lower rock mass produces passive extrusion and stress concentrated. Bending and uplifting deformation occur at the foot of the slope, and transverse bulging cracks also occur. The rock mass in this section is subjected to “passive” force, which corresponds to the “passive stress zone” in the mechanical mechanism. With the development of the landslide deformation, the original cracks in the back edge of the landslide extend to the depth, and the subsidence of the rock strata in the back edge intensively presents multi-stage platform.

Pore-water pressure increased driven by rainfall and melting snow infiltrated along the fissures and cracks can markedly augmented the geostatic gravity and reduced the shear strength of the slide belt. Thus, the hydrostatic pressure in the pulling surface at the back edge of the landslide simultaneously pushes the sliding body to slide along the potential sliding surface, which intensifies the destruction of the landslide rock mass. The thrust exerted by the upper rock mass on the lower rock mass intensified. The thrust effect of the upper rock mass enhances the bending deformation of the rock strata at the foot of the slope. The bending and rupture of the rock strata at the foot of the slope resulted in the shear failure of the rock mass at the bottom, which formed the shear slip surface. The shear slip surface traced the existing weak structures to the slope body, and finally made the slip surface expand and connect, thus forming a large-scale landslide with upper bedding and lower bedding cutting.

#### Triggering factors

**Fig. 17:**
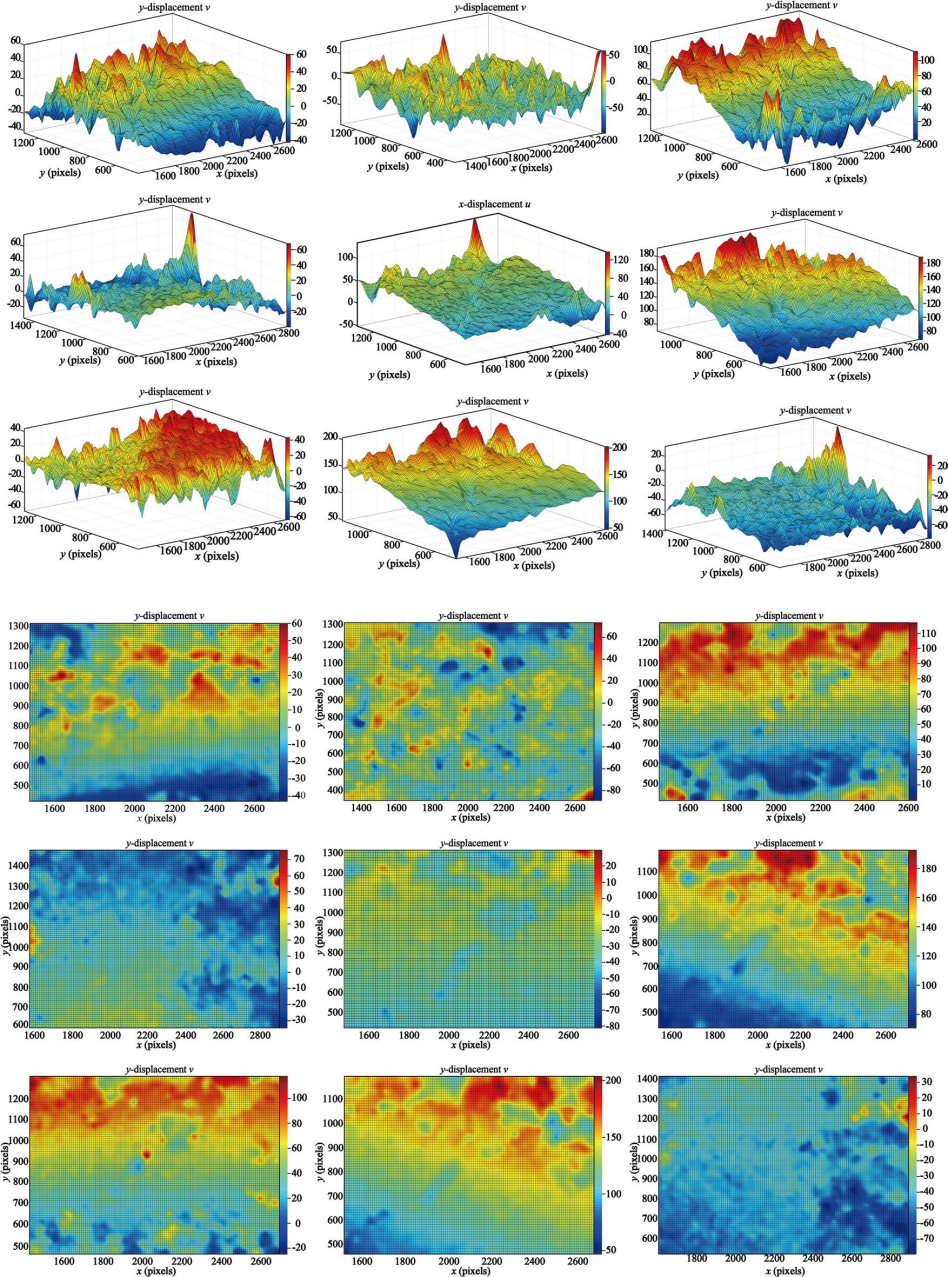
Signs of the landslide damage (**a**) main slope, (**b**) main scarp, (**c**, **d**), and (**e**) ground surface settlement, (**f** and **g**) working bench of trailing edge collapse, (**h** and **i**) cracks in the local surface expanded from 2014 ~ 20 (Fig. 17 was drawed and photographed by the 1st author Han Du).

**Fig. 18:**
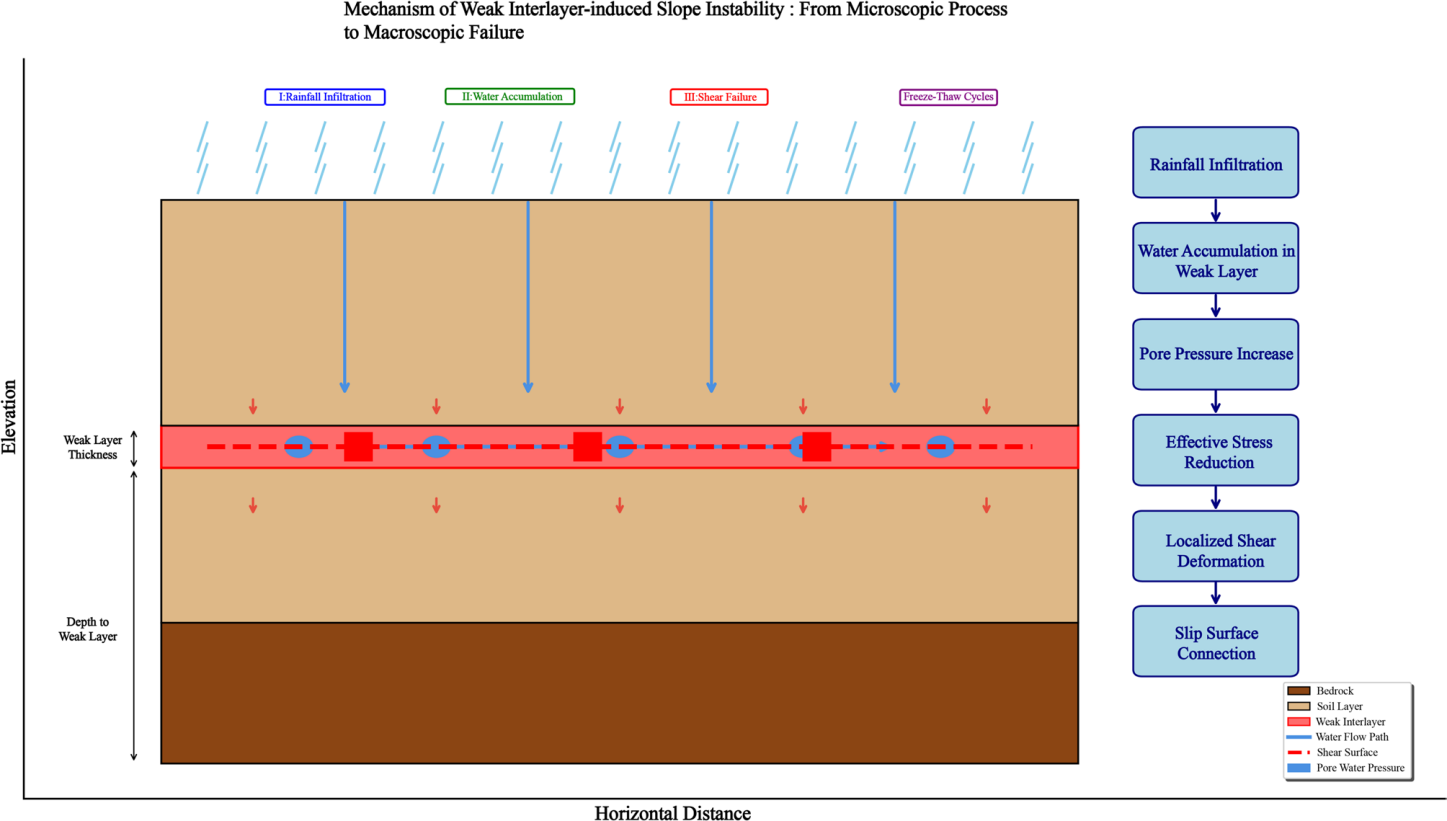
Mechanism of weak interlayer-induced slope instability from microscopic process to macroscopic failure.

The initiation of the slope failure derived from incorporation of internal factors as well as external factors (Regmi et al. 2017). Four primary factors are recommended to identify the type of the instability. In accordance with field investigation and the analysis of landslide deformation and slide process (as shown in (Fig. 17a–i)), the factor that triggered the landslide are sketched as below:

- Open-pit mining, which resulted in a pit with a current depth of about 110 m, has been one of the active factors contributing to the failure event. Generally, in an abiotic context, inclined slopes with manifold layered structures are naturally prone to have a natural tendency to develop a landslide. Secondly, mining activity, comprising excavation and blasting, also causes another anthropogenic forcing on deteriorating the rock mass quality and accelerating the speed of weathering. Surface excavation action for colliery construction (et al. minerals excavation, road maintaining, cut-slope excavation, drainage network ditching) can attenuate the stability of the slope as in situ stress are lower. Furthermore, the frequent mining activities can also markedly deteriorate the rock mass structures of slope body due to stress release. Additionally, tangential stress was distinct transferred owing to engineering slopes and multi-stage mining platform, and the shear stress concentrated at the toe of slope body. It can be also seen that there were several rock protruding and tension cracks in the landslide area. Moreover, after inquiring with the in-site staff, we learned that the eastern ramp in and out of the gully is the main transport route, all the trucks from detached transport vehicles are all with a load of more than 15 tonnes, this condition evidenced that heavy car at full horsepower uphill, empty car downhill brake, the vibration has a certain impact on the slope. Consequently, the landslide event was closely associated with open-pit excavation.
- Rainfall, spring torrential rainfall season, quantities of accumulated precipitation, and huge rainfall intensities were amalgamated with the most important hydro-pattern factor that causing the landslide. Due to a series of rainfall events, the slope erosion has been worsening with time, resulting in low cohesion and high mobility. Rainfall penetrated through the sand strata and concentrated in the mudstone strata, which seriously affected the stability of the slope. The accumulated rainfall of impending failure reached 310 mm. Because the ecological water-holding capacity of Baorixile open-pit mine is drawdown by human activities, the direct seepage of torrential rainfall and the infiltration of the slope surface make the slope body tend to be saturated. The effects of rainfall action include (1) reducing the mechanical parameters (cohesion and internal friction angle) of stratum of slope body; (2) raising the groundwater level, making the mechanical parameters of the sliding zone of the foundation cover interface drop sharply, reducing the anti-sliding force of landslide; (3) increasing the bulk density of rock masses, increasing the sliding force of landslide mass. (4) the dynamic water pressure of torrential rainfall causes the loss of fine-grained soil mass in the outcrop layer washed away from bed deposits; In consequence, the slope structure is loose and cohesion is reduced, which is more conductive to rainfall infiltration. Additionally, rainfall weaken the physical and mechanical properties of the slope body likewise.
- Weak intercalation strata effect, the weak intercalation layer of open-pit mine is a relative concept, and the properties of the weak intercalation layer vary greatly in different rock masses (in Fig. 18). On the basis of large numbers of consulting literatures to the analytic of the influencing factors of slope slumping in open-pit mines and the definition and analysis of the weak strata, we combined with the stratigraphic lithology of this area and the test results and went through the relevant information and engineering rock classification standards to determine the criterion of weak intercalation strata is: (1) Structural characteristics, whether the microstructure is similar to clay; (2) Water characteristics: easy to soften in water, completely disintegration time less than 6 h with weak permeability; (3) Hydrophilic mineral content, kaolinite more than 15%, illite more than 8%, montmorillonite more than 6%; (4) Physical and mechanical indicators: moisture content larger than 10%, clay content is greater than 40% ~ 50%, natural density is less than 2 g/cm^3^; Searing strength index: *φ* < 20°, *c* < 60 kPa. The combined effects—(i) low intrinsic shear strength, (ii) hydrologic lubrication and effective-stress reduction during rainfall, and (iii) freeze–thaw degradation—favor shear localization along the interlayer, thereby preconditioning Stage II–III deformation and ultimately facilitating Stage IV sliding/toppling.

Through the differential thermal analysis of the clay minerals in the coal seam of the second mining area shows that the content of kaolinite is 6%-12%, elite 4%-12% and montmorillonite 4%-14%. Differential thermal analysis (DTA) tests show that the clay contains high hydrophilic montmorillonite and elite (hydromica). The water content of soft interlayer is 50% ~ 55%, the shear strength is very low, and the internal friction angle (*φ*) is less than 5°. According to the strata that have been revealed, the whole area of the second mining area is developed with weak interlayer, which is located 10 m ~ 14 m below the coal seam roof and is 2 cm ~ 5 cm thick. The weak interlayer is brown, black brown and soft plastic. According to the laboratory test, its shear strength is very low. The weak strata plays a decisive role in the landslide event. On the one hand, the mechanical parameters of the weak strata are very low; on the other hand, the weak layer forms a good water accumulation condition. The interaction of the two aspects indicates that the weak strata is of the triggering factor of this landslide event.

Freezing and thawing effect, Baorixile open-pit mine is located in the transitional area between high latitude seasonal frozen soil area and permafrost. The lowest temperature in winter is below -40 ℃, the effect of frost heave ablation on slope is obvious. The slope body is subjected to the periodic freezing an thawing cycle year after year, and the environment of freezing and thawing make the slope body are predisposed to occur crumble, which has become one of the important triggering factor. The main performance can be attributed to two aspects as follows: Under the condition of negative temperature in winter season, the water in the slope crystallized, which freezes the slope soil, produces uneven frost heave, and destroys the original structure of the slope soil; in the spring thawing period, the frozen soil on the surface of the slope begins to melt, and the slope instability occurs under the action of the soil’s own gravity, resulting in spring thawing landslide.

## Conclusions

1. Landslide this work links the climatic forcing–mining activity interplay to a validated four-stage hazard chain and, critically, converts it into risk-zoning that maps to differentiated controls across the rear tension cracking, top detachment, middle sliding–subsidence, and anterior colluvium domains. The Balzhikher landslide event was precipitated by the combined effects of freeze–thaw cycles and mining operations, which compromised slope stability. In complicated geological settings, weak interlayers, including mudstone and carbonaceous mudstone, are critical variables contributing to landslide occurrences influenced by mining activity and climate change. Variations in climatic circumstances, including recurrent freeze–thaw cycles, facilitate the infiltration of moisture from the geological layer into the rock and soil bulk, hence exacerbating slope instability.
2. We provide an actionable basis for early warning by defining percentile-based AL-DIC thresholds anchored by GNSS/crack time series and rainfall episodes, enabling site-specific watch/warning settings for slope management. The enduring effects of climatic variables and mining operations on the geological strata necessitate systematic surveillance and data analysis to ascertain potential landslide hazards. By integrating climate, geological, and mining activity data, a more precise landslide prediction model can be developed to detect hazardous slopes proactively, hence facilitating early warning and disaster mitigation strategies.
3. Landslide the findings translate into prioritized prevention and control measures—drainage optimization and bench re-grading in uplift–subsidence belts, reinforcement along weak interlayers, targeted unloading at the run-out front, and operations de-risking during elevated-strain stages. The subsequent approaches for prevention and control are recommended:
  1. Slope reinforcement in regions with weak interlayers can significantly mitigate the incidence of landslides. In regions where freeze–thaw cycles are significant, employing specialised slope stabilisation measures can avert structural collapse caused by water accumulation.
  2. Enhancement of the drainage system: Given that the fluctuations of groundwater and surface water significantly contribute to landslide occurrences, it is imperative to enhance the drainage system inside the mining area. In regions characterised by significant precipitation and recurrent freeze–thaw cycles, an effective drainage system can mitigate moisture accumulation and hence diminish the risk of landslides.
4. A low-cost, transferable toolkit—fixed-camera AL-DIC plus periodic UAV surveys—is presented, enabling mines with limited instrumentation to transition from point/line measurements to full-field diagnostics and improving early-warning reliability. LANDSLIDE This approach can monitor slope stability in real time and detect potential landslide threats by a complete analysis of remote sensing imagery and on-site monitoring data in mining regions. The ongoing advancement of remote sensing technologies and data collection techniques can enhance the precision of landslide predictions and offer prompt early warnings for mining regions.
5. By integrating environmental data (rainfall/freeze–thaw) with remote-sensing products and on-site logs, the framework strengthens decision-readiness for mine-scale early-warning systems and supports production scheduling during adverse conditions. Landslide integrating environmental data, including meteorological information and groundwater level fluctuations, with remote sensing data enhances the accuracy of landslide predictions. This novel technology offers a theoretical framework for the development of landslide prevention and mitigation strategies in mining regions, enhancing safety and decreasing the frequency of landslide incidents in actual implementations.

## Data Availability

The data that support the findings of this study are available from the corresponding author upon reasonable request.
